# Supplementary material for: Trade-offs between individual and ensemble forecasts of an emerging infectious disease
Source: Nat Commun. 2021 Sep 10;12:5379. doi: 10.1038/s41467-021-25695-0 (PMC8433472; doi:10.1038/s41467-021-25695-0)
Supplement: Supplementary file 1 — Supplementary Information [file 41467_2021_25695_MOESM1_ESM.pdf]

Supplementary Information

Trade-offs between individual and ensemble forecasts of an emerging infectious disease

Oidtman *et al.*

Supplementary methods

Priors on parameters common to all models

In each model that we considered, we iteratively estimated the reporting probability (ρ), dispersion parameter of the negative binomial distribution (ϕ), R multiplier (k), and the timing of the first infection (ι). When possible, we leveraged previous estimates of parameters to inform prior distributions for model parameters. In some instances, we used dengue-specific parameter estimates as priors for Zika-specific parameters [1].

For the reporting probability (ρ), we assumed a mean of 0.2 and a variance of 0.05. Although this mean and variance are not directly informed by an empirical study of Zika reporting, they are in line with what we would expect for dengue [2, 3] and Zika [4]. We assumed that ρ was a beta random variable and, using the method of moments, we specified a prior distribution for ρ such that

$$\rho \sim \text{beta}(0.44, 1.76) \tag{10}$$

which resulted in mean and variance consistent with our prior assumptions.

The dispersion parameter of the negative binomial distribution accounts for variability in case reporting beyond that captured by ρ. Lower values of the dispersion parameter indicate overdispersion, such that variability in cases cannot be explained by a single rate of case incidence, as would be generated by a Poisson distribution with rate $\rho I_{d,t}$. Given the likelihood of variation in the reporting probability over the course of the epidemic [5] and across departments [6], we specified a uniform prior for ϕ,

$$\phi \sim \text{uniform}(0, 1), \tag{11}$$

which resulted in a level of overdispersion in reporting equal to at least a geometric distribution ($\phi = 1$) but potentially greater ($\phi < 1$).

To relate the transmission coefficient ($\beta_{d,t}$) to environmentally driven descriptions of the reproduction number ($R_{d,t}$), we used a multiplier (k) that applied to both the static and dynamic models of R. We specified a gamma prior distribution,

$$k \sim \text{gamma}(2.25, 0.75), \tag{12}$$

the parameters of which were chosen by moment matching to result in a mean of three and a variance of two.

To seed the Zika epidemic in Colombia, we assumed that undetected transmission could have been occurring at any time throughout the first 34 weeks of 2015. For reference, the first case was not reported until August 9, 2015 (week 35). Thus, we specified a uniform prior for the date of the initial introduction (ι_1) between weeks 1 and 34 of 2015. We assumed that the location of the first introduction (l_1) could have been any of the departments in Colombia with equal prior probability.

Assumptions about human mobility

Spatially coupled models

For the spatially coupled models, we assumed that transmission was coupled across departments by human mobility. In these models, we informed the spatial connectivity matrix in three ways: i) aggregated mobility patterns extracted from mobile phone call detail records (CDRs), ii) a gravity model, or iii) a radiation model. In

the gravity and radiation models, $T_{i,j}$ is defined as the total number of trips from department i to department j. This takes the form $T_{i,j} = c\frac{N_i^\alpha N_j^\beta}{d_{i,j}^\gamma}$ in the gravity model and $T_{i,j} = T_i \frac{N_i N_j}{(N_i+s_{i,j})(N_i+N_j+s_{i,j})}$ in the radiation model, where N_i and N_j are population sizes at the origin i and destination j, $d_{i,j}^\gamma$ is the distance between i and j, $s_{i,j}$ is the total population within radius $d_{i,j}$ from i, and T_i is the total number of individuals who make a trip. The parameters c, α, β, and γ were fitted to CDRs from Spain and validated in West Africa [7]. All three mobility models were row-normalized to correspond to the proportion of time spent by residents of department i in department j.

Spatially uncoupled models

For the spatially uncoupled models, we assumed that each department's epidemic occurred independently of all other departments. We used the same prior distributions as described above for ρ, ϕ, k for each department. Under this assumption, we did not include a parameter for the location of the initial introduction into Colombia, as we instead were concerned with the initial introduction into that department. It was still necessary to specify the timing of that introduction and, for models that considered it, the timing of a second introduction. Following the rationale that undetected transmission could have occurred for up to 34 weeks prior to the first reported case in a given department, we assumed an even prior for the timing of the introduction(s) into a given department.

Assumptions about environmental drivers of transmission

Dynamic model

The environmentally driven component of $\beta_{d,t}$ for the dynamic model, $\tilde{R}_{d,t}(T_{d,t}, OP_{d,t}|c, \psi, \alpha, v)$, was defined as the product of two functions: one that depends on $T_{d,t}$ and one that depends on $OP_{d,t}$.

The function of $OP_{d,t}$ was defined as $c(-\log(1 - OP_{d,t}))$, which converts occurrence probability into an expectation of mosquito abundance [8]. The constant c scales this expectation to account for uncertainty in its magnitude, which we estimated and assumed to have a gamma-distributed prior with a shape parameter of 16 and a rate parameter of 0.07. This choice of prior resulted in average $\tilde{R}_{d,t}$ being equal to one when evaluated at the mean values of the prior for c.

The function of $T_{d,t}$ was based on a temperature-dependent description of the basic reproduction number by Mordecai et al. [9]. Specifically, we used the version of that model based on Briere functions for parameters that were not otherwise accounted for in estimates of mosquito occurrence probability [10]. Those parameters include the mosquito biting rate (a), mosquito-to-human transmission probability (b), human-to-mosquito transmission probability (c), average adult mosquito lifespan (lf), and parasite development rate (PDR). Those functions combine to form the temperature-dependent component of $\tilde{R}_{d,t}$,

$$a(T_{d,t})b(T_{d,t})c(T_{d,t})e^{-1/(lf(T_{d,t})PDR(T_{d,t}))}PDR(T_{d,t}). \tag{13}$$

We did not include other parameters from Mordecai et al. [9] related to mosquitoes, such as immature development rate and egg-to-adult survival rate, as the effects of those parameters were accounted for in estimates of $OP_{d,t}$ from Kraemer et al. [10].

To reduce the number of parameters that we needed to estimate, we worked with a simplified description of the temperature curves produced by eq. (13) (Fig. 5). To do so, we took random draws from the posterior distribution of parameters from Mordecai et al. [9], computed functions of temperature according to eq. (13), and fitted parameters of a skew normal distribution to those curves by least squares. The skew normal distribution has three parameters—location (ψ), scale (α), shape (υ)—that together control the mean, variance, and skew of the distribution, which is sufficient to approximate the uncertainty in posterior predictions of the temperature curves described by eq. (13). We then took the mean and covariance across those estimates of ψ, α, and υ to describe their variation with a multivariate normal distribution, which was the prior distribution we used for those parameters at the first step of our particle filter.

Static model

The environmentally driven component of β_d for the dynamic model, $\bar{R}_d(T_{d,\vec{t}}, OP_d, PPP_d)$, was defined as the product of three functions: one that depends on $T_{d,\vec{t}}$, one that depends on OP_d, and one that depends on PPP_d. We used values of $\bar{R}_d$ at the 5 km x 5 km grid cell level as calculated by Perkins et al. [8]. To aggregate them to the department level, we took a population-weighted mean of $\bar{R}_d$ across 5 km x 5 km grid cells within each department. Although we made no other modifications to the calculation of $\bar{R}_d$, we summarize the methodology used by Perkins et al. [8] in the interest of comparability with the dynamic model.

The function of OP_d was defined as $-\log(1 - OP_d))$, which was the same procedure used to convert occurrence probability into an expectation of mosquito abundance as used in the dynamic model. For the static model, however, we followed Perkins et al. [8] and relied on a description of occurrence probability that was not defined on a time-varying basis [10].

Rather than estimate a scaling parameter like c in the dynamic model, we relied on a scaling parameter defined as a function of PPP_d by Perkins et al. [8]. This function took the form of a monotonically decreasing, cubic spline function estimated with a shape-constrained additive model. The data that informed this estimate of both the relationship with PPP_d and the magnitude of $\bar{R}_d$ were outbreak sizes of 12 chikungunya outbreaks and one Zika outbreak [8] that occurred prior to the ZIKV invasion of the Americas.

The function of $T_{d,\vec{t}}$ was based on a temperature-dependent description of the basic reproduction number that includes temperature-dependent descriptions of mosquito mortality (μ) [11] and the extrinsic incubation period (n) [12]. Those functions contribute to an expression for monthly contributions to $R_{d,t}$,

$$\frac{bca^2 e^{-\mu(T_{d,t})n(T_{d,t})}}{\mu(T_{d,t})r}, \tag{14}$$

along with constants that represent the mosquito-to-human transmission probability (b), human-to-mosquito transmission probability (c), mosquito biting rate (a), and rate of recovery from human infection (r). For each department d, we took the average of the six largest values of eq. (14) as the contribution of $T_{d,\vec{t}}$ to $\bar{R}_d$.

Assumptions about introduction events

One-introduction models

Each of the one-introduction models assumed infections were seeded at one point in time (ι_1) in one location (l_1), as specified in the general model parameters section of the Supplementary Methods.

Two-introduction models

Each of the two-introduction models made similar assumptions about ι_1 and l_1 for the first introduction. For these models, we additionally specified the timing (ι_2) and location (l_2) of the second introduction events, for which we assumed even priors. In reality, there were likely more than only one or two introductions throughout the epidemic, but genomic epidemiology suggests the majority of local transmission resulted from only one or two introductions [13].

Supplementary tables

Symbol	Definition	Class
ρ	Reporting probability	Estimated parameter
ϕ	Overdispersion in reporting	Estimated parameter
k	$R_{d,t}$ multiplier	Estimated parameter
ι_1	Timing of first infection	Estimated parameter
ι_2	Timing of second infection	Estimated parameter
l_1	Location of first infection	Estimated parameter
l_2	Location of second infection	Estimated parameter
c	Dynamic R scalar	Estimated parameter
ψ	Location for skew normal	Estimated parameter
α	Scale for skew normal	Estimated parameter
υ	Shape for skew normal	Estimated parameter
θ	Parameter set	Set of estimated parameters
$I_{d,t}$	Simulated infections	State variable
$I'_{d,t}$	Redistributed infections	State variable
$I''_{d,t}$	Effective number of infections	State variable
$C_{d,t}$	Simulated cases	State variable
$y_{d,t}$	Observed data	Data
d	Department index	Data
t	Time; $t = 0, ..., T$	Data
n	Particle index; $n = 1, ..., N$	Data
η	Timing of first reported case	Data

Supplementary Table 1: Mathematical symbols for parameters, state variables, and data with their respective meanings.

Supplementary figures

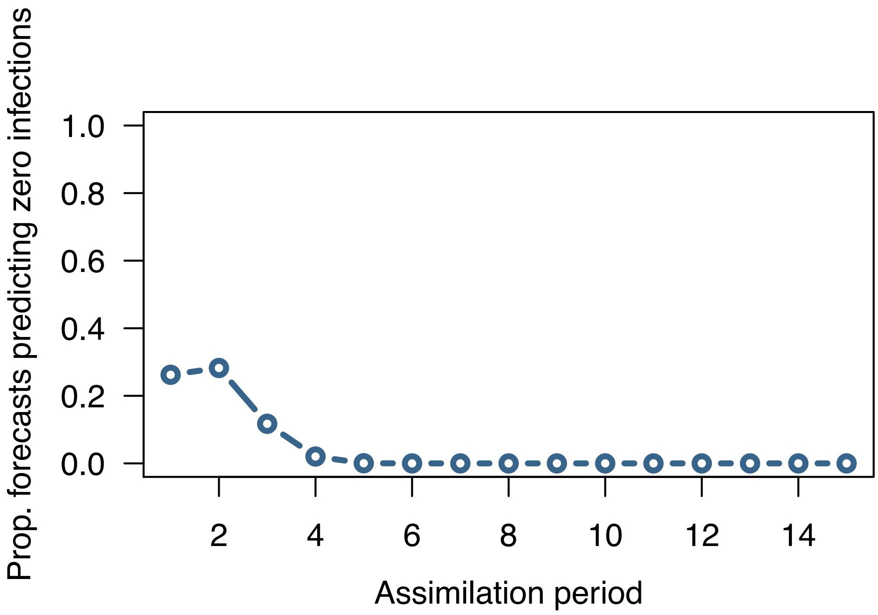

Supplementary Figure 1: **Proportion of forecast trajectories predicting zero infections.** Forecast trajectories are specific to each particle and this example is from the model using CDR-derived mobility data, static R, and one importation event, corresponding to Fig. 2.

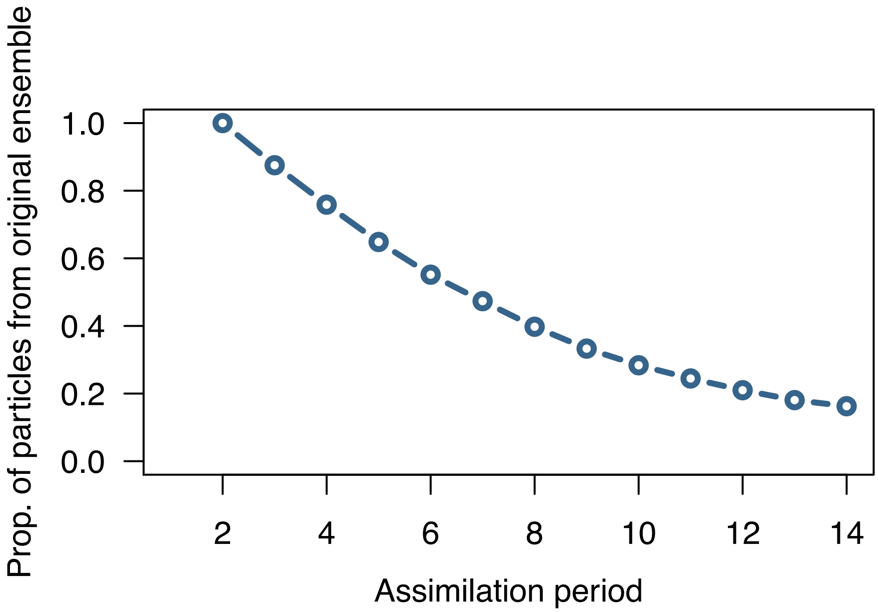

Supplementary Figure 2: **Proportion of particles that remain from the original particle ensemble present in the retained ensemble at each assimilation period.** This example is from the model using CDR-derived mobility data, static R, and one importation event, corresponding to Fig. 1.

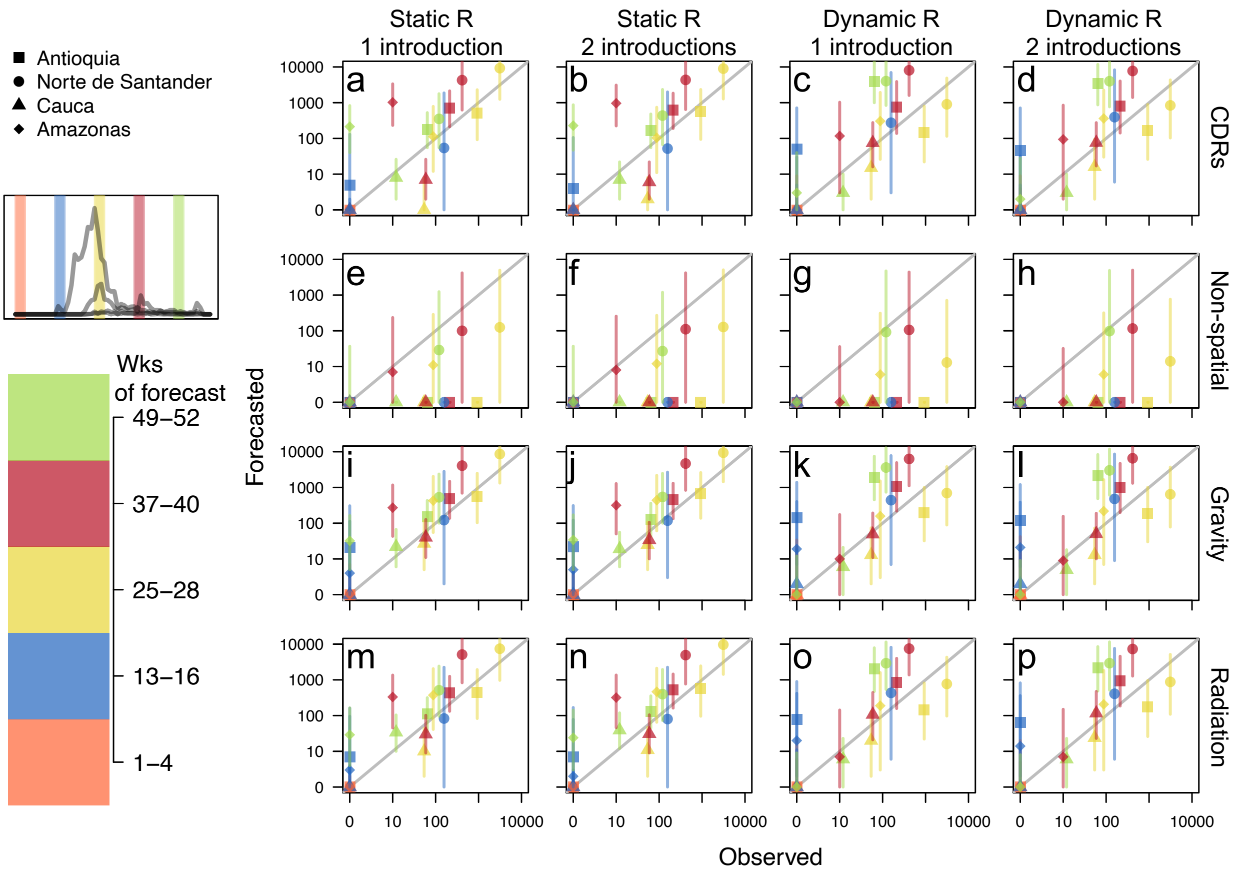

Supplementary Figure 3: **Cumulative observed versus forecasted incidence at 4-week ahead intervals for each individual model for Antioquia, Norte de Satander, Cauca, and Amazonas at five points throughout the epidemic.** *a-p*. Each plot represents a different model, with model features labels on rows and columns. Plotted departments reflect differences in population, epidemic size, and geographic regions of Colombia and are denoted by point type. Point shape denotes department. Point color indicates time at which forecasts were generated and is visually denoted in inset plot and color bar. 1-, 2-, 3-, and 4-week ahead forecasts and observed incidence were aggregated for ease of comparison. Points are the median values and lines are the 50% credible interval, which were calculated from 2,000 draws from the posterior fit of the mode. 1:1 line is in grey.

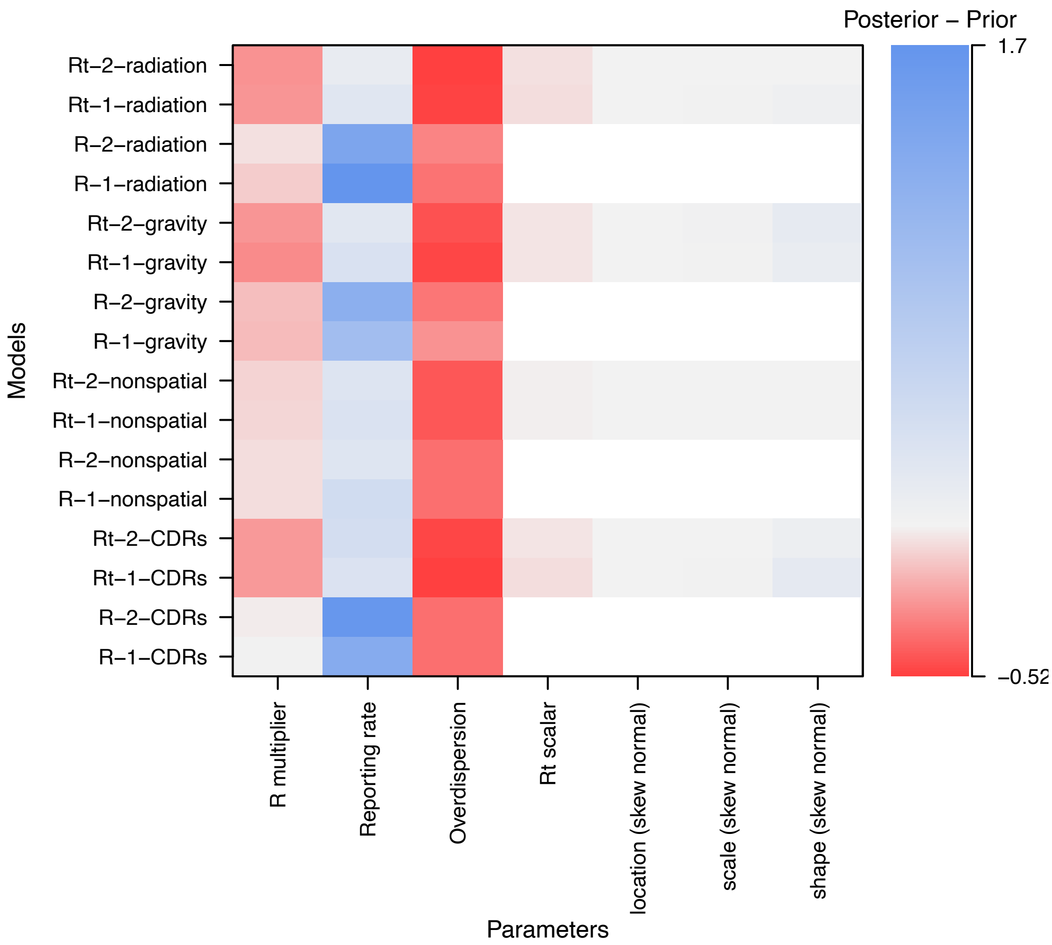

Supplementary Figure 4: **Relative difference between the prior estimates of the parameters and the posterior estimates at the final time point in the epidemic.** Parameters include the R multiplier (k), reporting rate (ρ), overdispersion parameter (ϕ), R_t scalar (c), and the location (ψ), shape (α), and scale (ν) parameters for the skew normal distribution. Blue indicates posterior estimates were higher than prior estimates, red indicates posterior estimates were lower than prior estimates, and grey indicates no difference. Areas in white indicate the corresponding model does not use that parameter. To calculate the relative difference, we subtracted the prior estimates of parameters from the posterior estimates of parameters and divided the difference by the prior to standardize over different parameter magnitudes. For comparison purposes, we left out the initial timing and initial location of ZIKV introduction parameters.

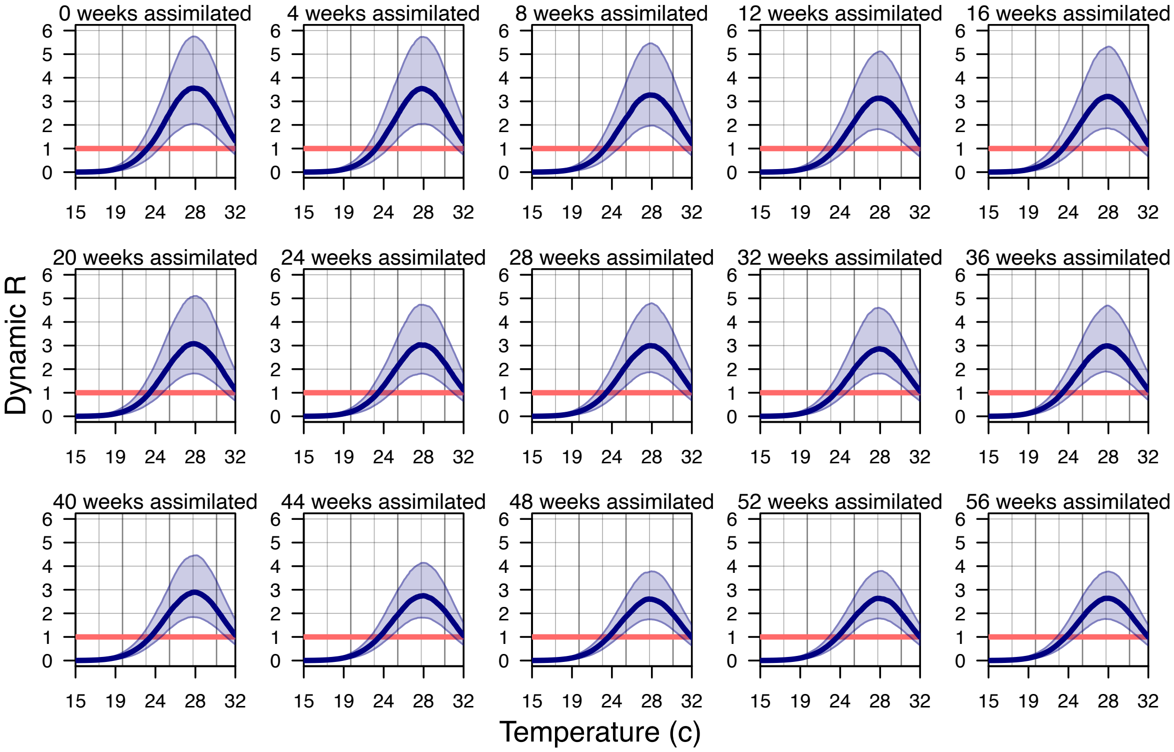

Supplementary Figure 5: **Estimates of R at each assimilation week across temperatures for average mosquito occurrence probability in Colombia.** Blue bands indicate 95% credible interval, with think navy line indicating the median estimate of R. Horizontal red line indicates $R = 1$.

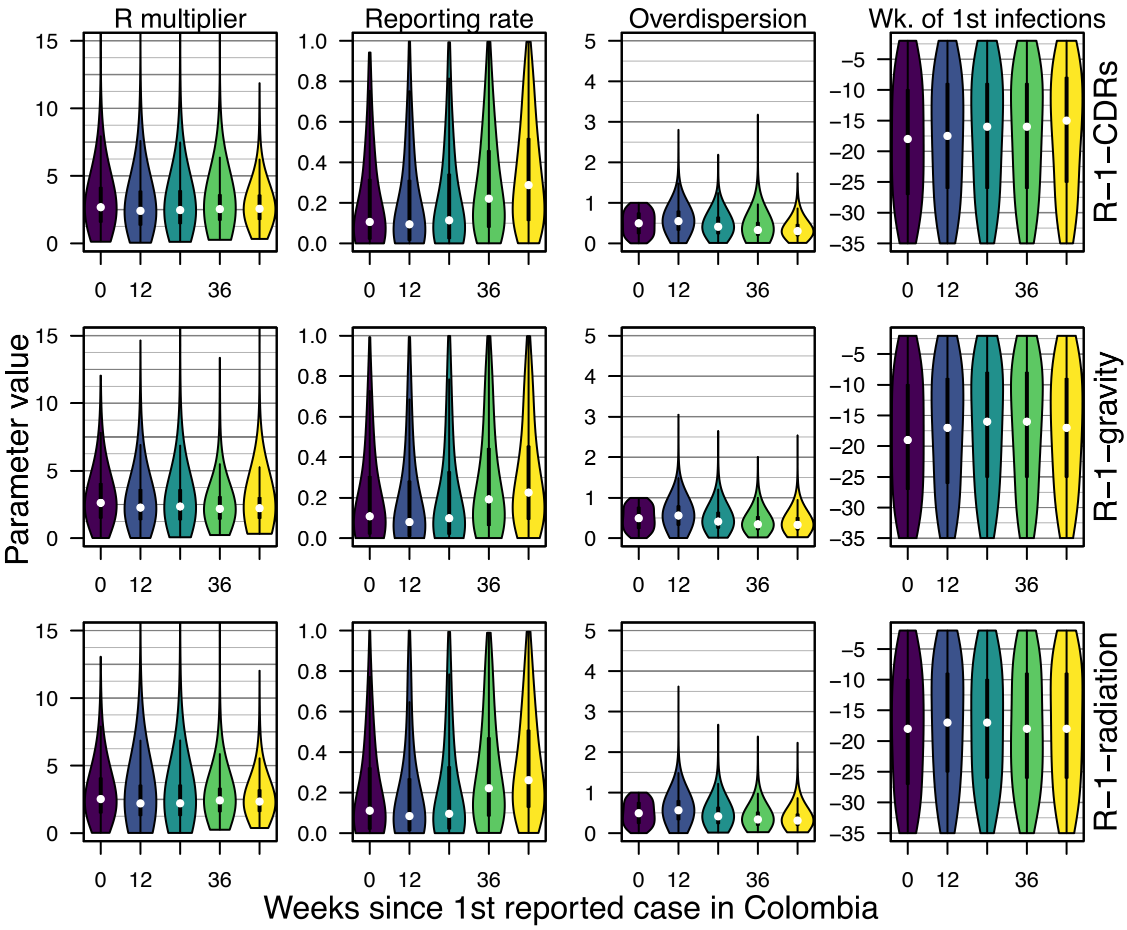

Supplementary Figure 6: **Posterior distributions of model parameters for the spatially coupled models with a static R at five different points in time.** The R multiplier (k), reporting rate (ρ), the overdispersion parameter (ϕ), and the timing of the first importations (ι_1) were model parameters represented in each model, although we are showing the posterior distribution only for a subset of the models. Violin plots are colored by time at which the forecasts were made, and correspond to time points in Fig. 3 and Fig. 23, 10

.

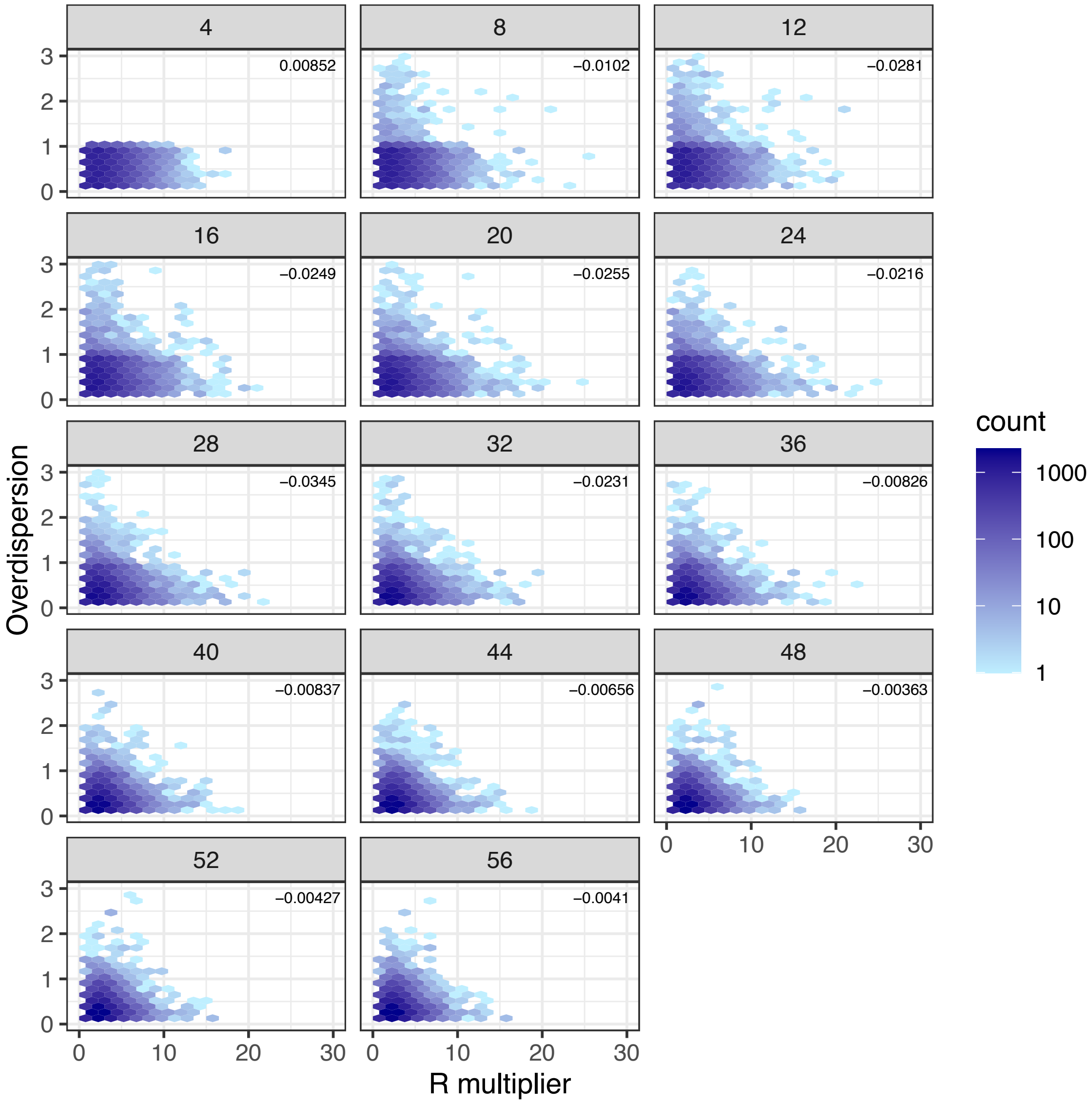

Supplementary Figure 7: R_d **multiplier versus the overdispersion parameter in the negative binomial distribution.** The overdispersion parameter in the negative binomial distribution represents the variability in the reporting probability. Pearson's correlation coefficient for the two parameters within a parameter set is listed in the top right corner. This example is from the model using CDR-derived mobility data, static R, and one importation event, corresponding to Fig. 1.

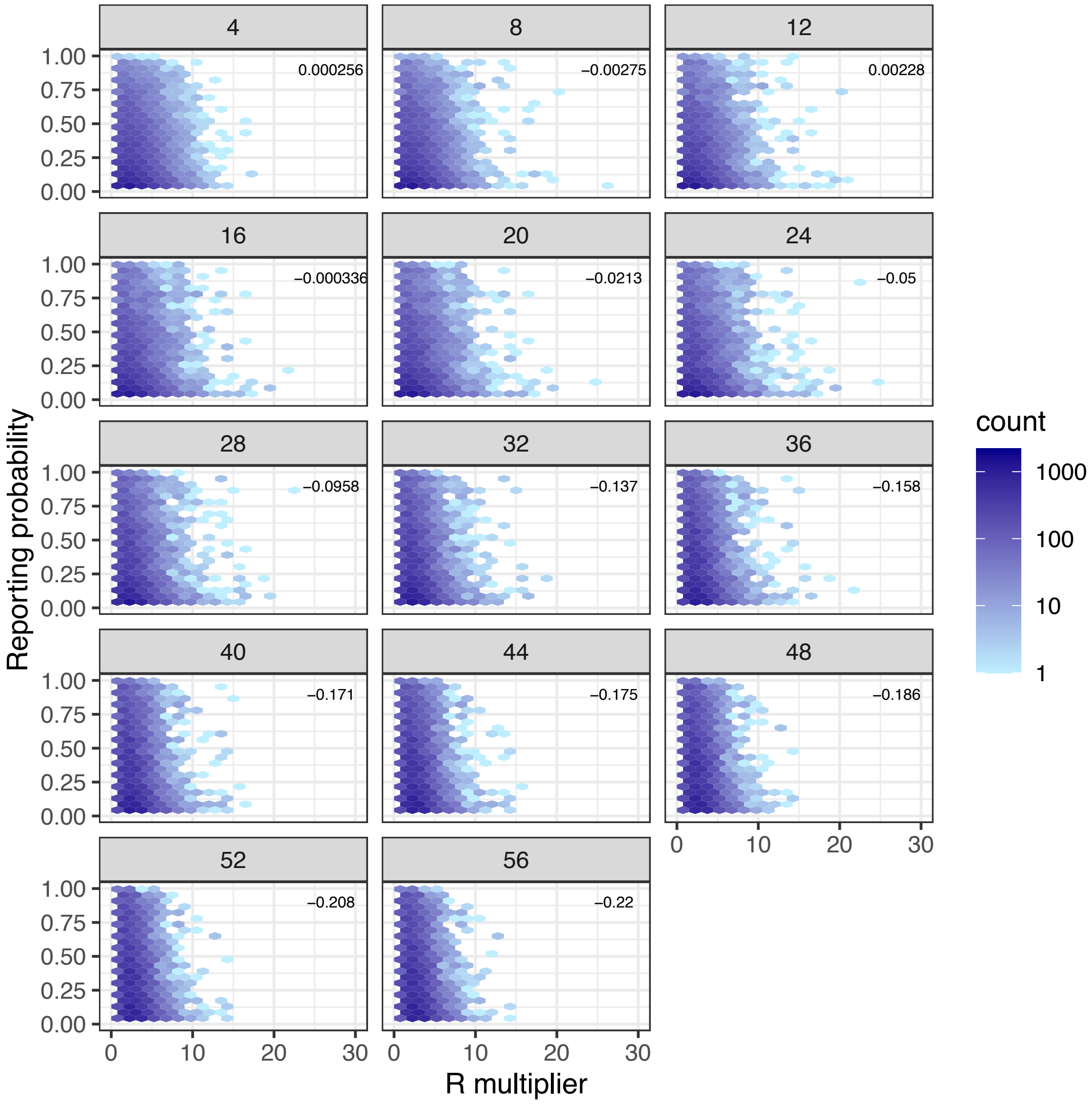

Supplementary Figure 8: R_d **multiplier versus the reporting probability.** Pearson's correlation coefficient for the two parameters within a parameter set is listed in the top right corner. This example is from the model using CDR-derived mobility data, static R, and one importation event, corresponding to Fig. 1.

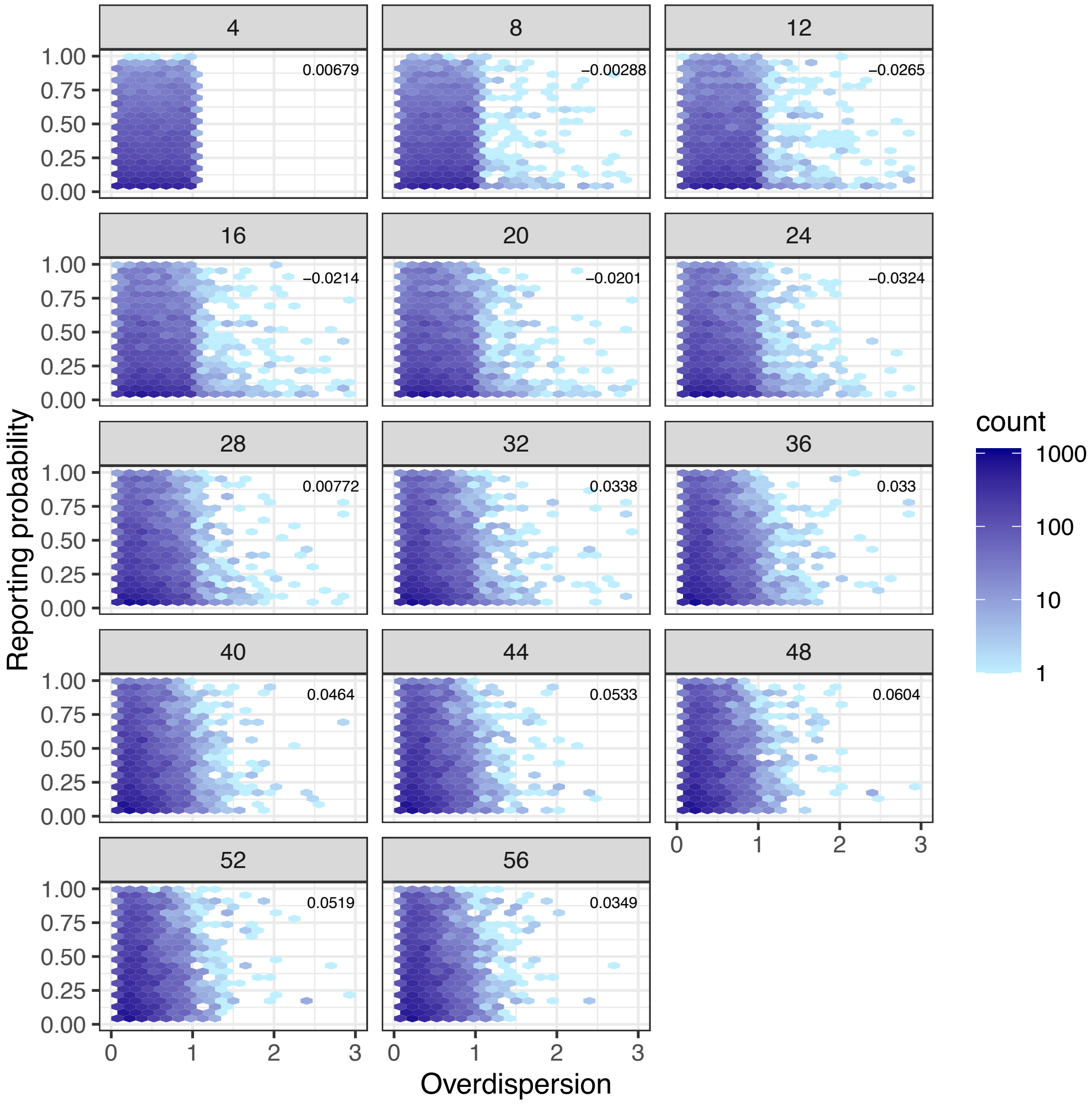

Supplementary Figure 9: **The negative binomial overdispersion parameter versus the reporting probability for one example model at each assimilation period.** The overdispersion parameter in the negative binomial distribution represents the variability in the reporting probability. Pearson's correlation coefficient for the two parameters within a parameter set is listed in the top right corner. This example is from the model using CDR-derived mobility data, static R, and one importation event, corresponding to Fig. 1.

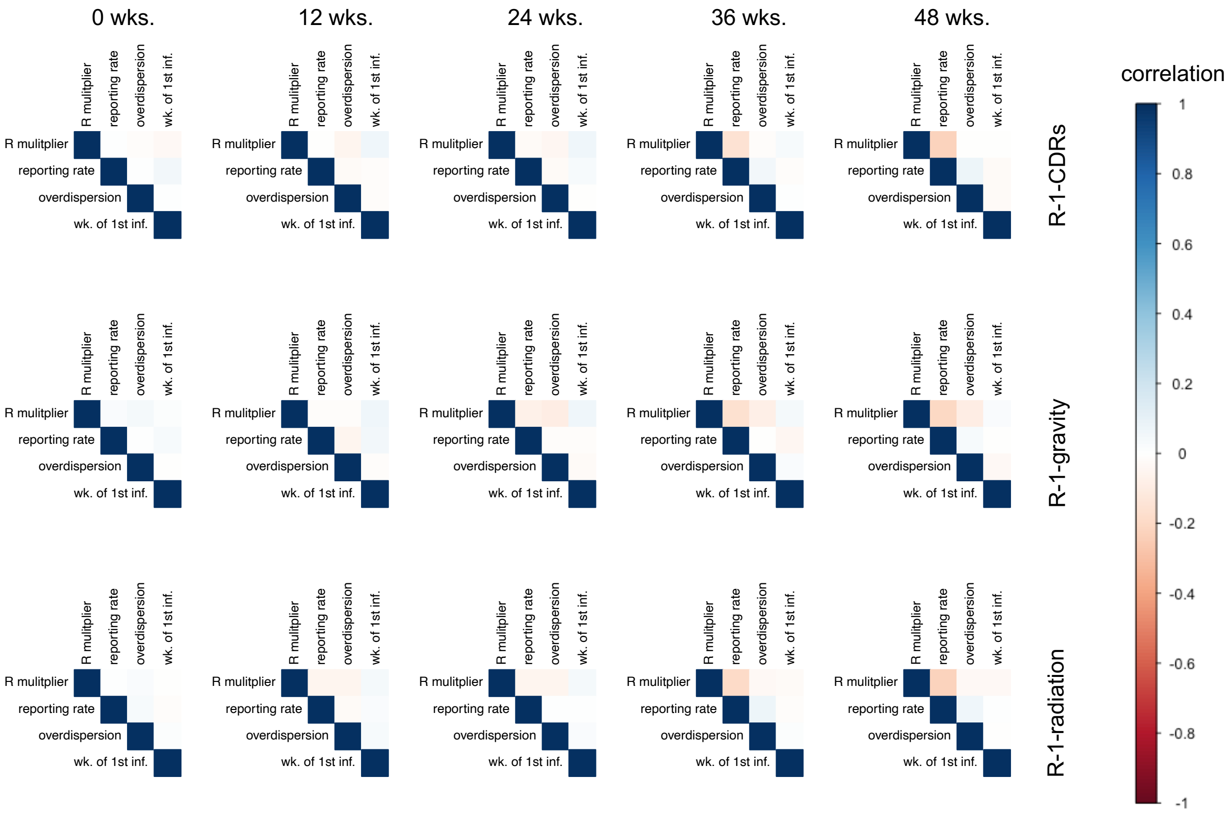

Supplementary Figure 10: **Correlation of model parameters across within a parameter set through time for the spatially coupled models with a static R at five different points in time.** Parameters include, the R multiplier (k), reporting rate (ρ), the overdispersion parameter (ϕ), and the timing of the first importations (ι_1) as they were represented in each model, although we are showing the correlations only for a subset of the models. Time points shown here correspond to those time points in Fig. 3 and Fig. 23, 6.

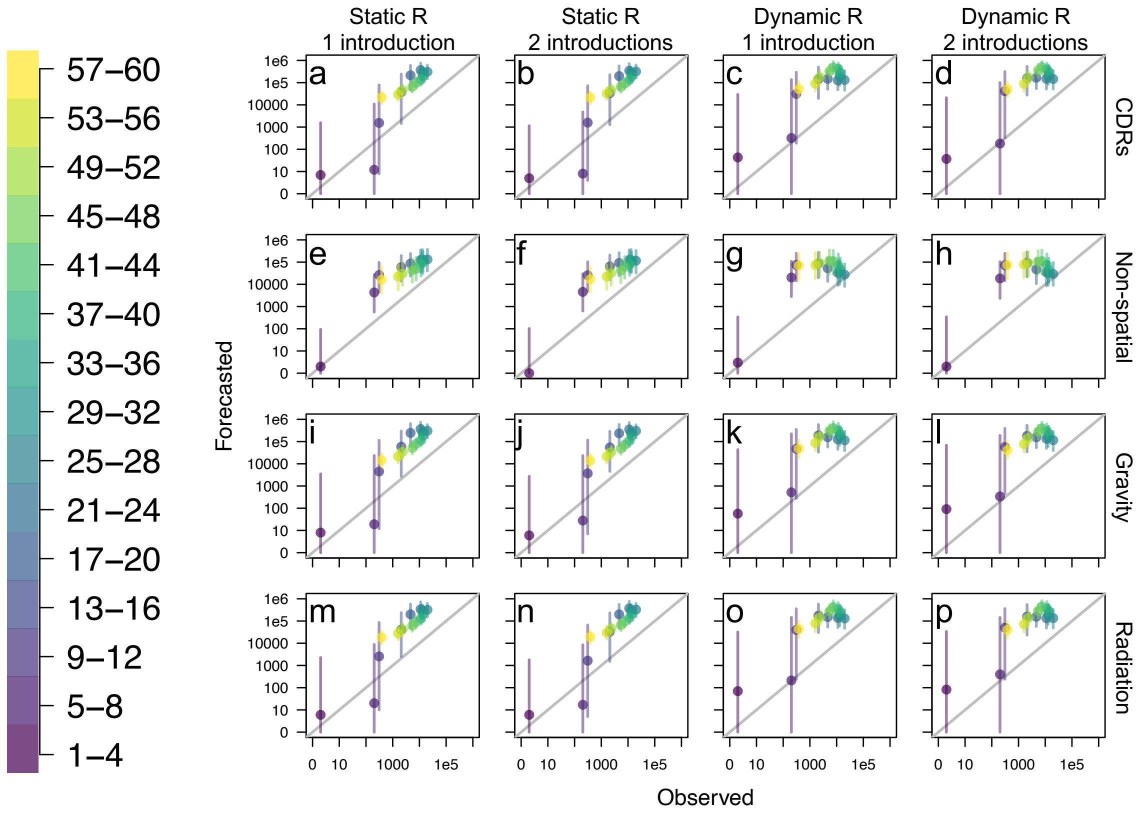

Supplementary Figure 11: **Cumulative observed versus forecasted incidence at 1-, 2-, 3-, 4- week ahead intervals for each individual model aggregated nationally at each point throughout the epidemic.** *a-p.* Each plot represents a different model, with model feature labels on rows and columns. Point color indicates time at which forecasts were generated. Points are the median values and lines are the 50% credible interval, which were calculated from 2,000 draws from the posterior fit of the model. 1:1 line is in grey.

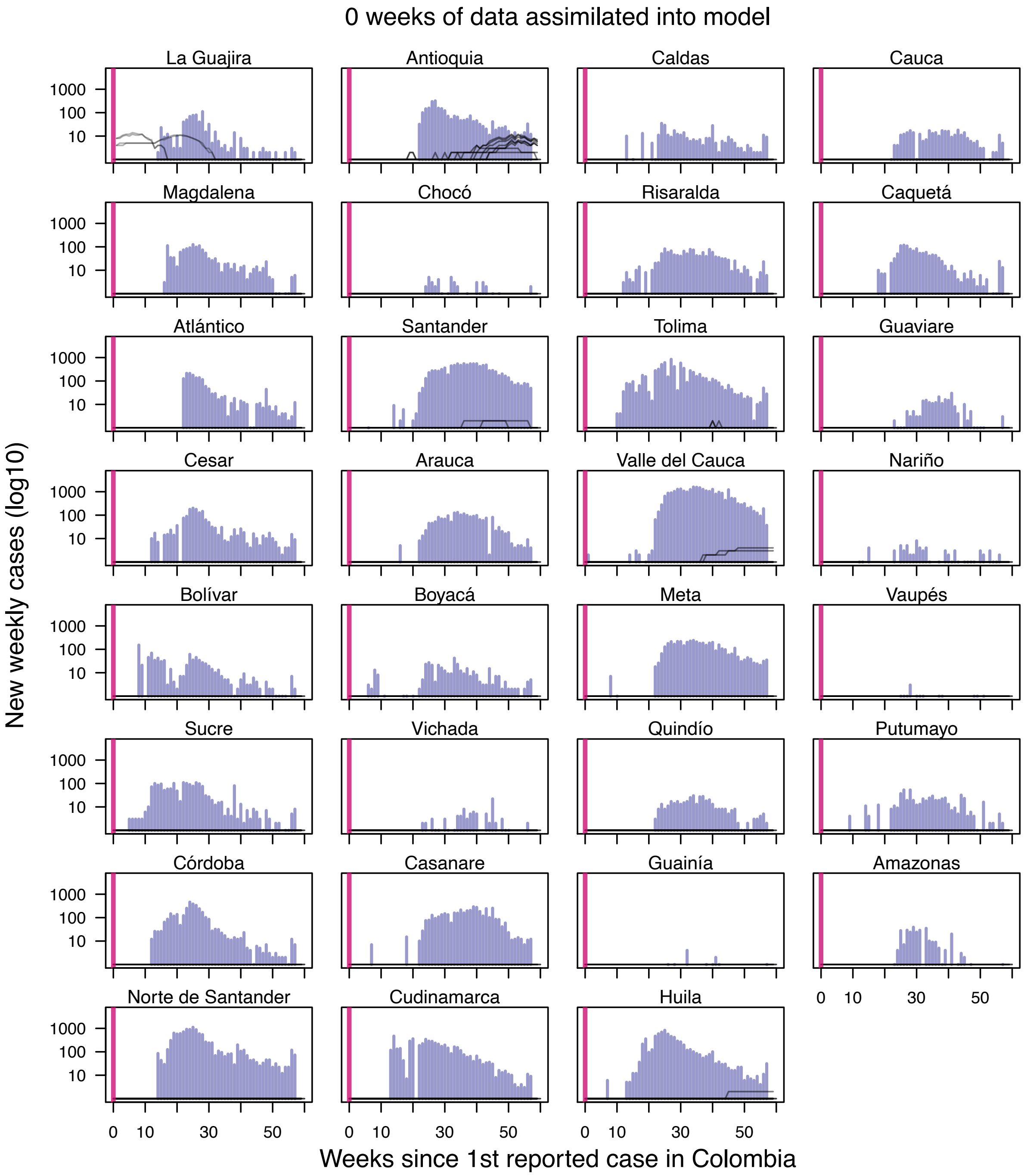

Supplementary Figure 12: **Observed incidence with initial median forecast for each of the 16 models with no weeks of data yet assimilated forecasting models.** Navy bars indicate Zika incidence data at weekly time interval [14]. Vertical line indicates the point at which the forecast was made, with data to the left of the line assimilated into the model. Forecasts to the right of the vertical line change as more data is assimilated into the model, while model fits to the left of the vertical line do not change. With the forecasts generally being at zero cases in the majority of the departments, we see that models were unlikely to forecast an epidemic to occur when no data was yet to be assimilated.

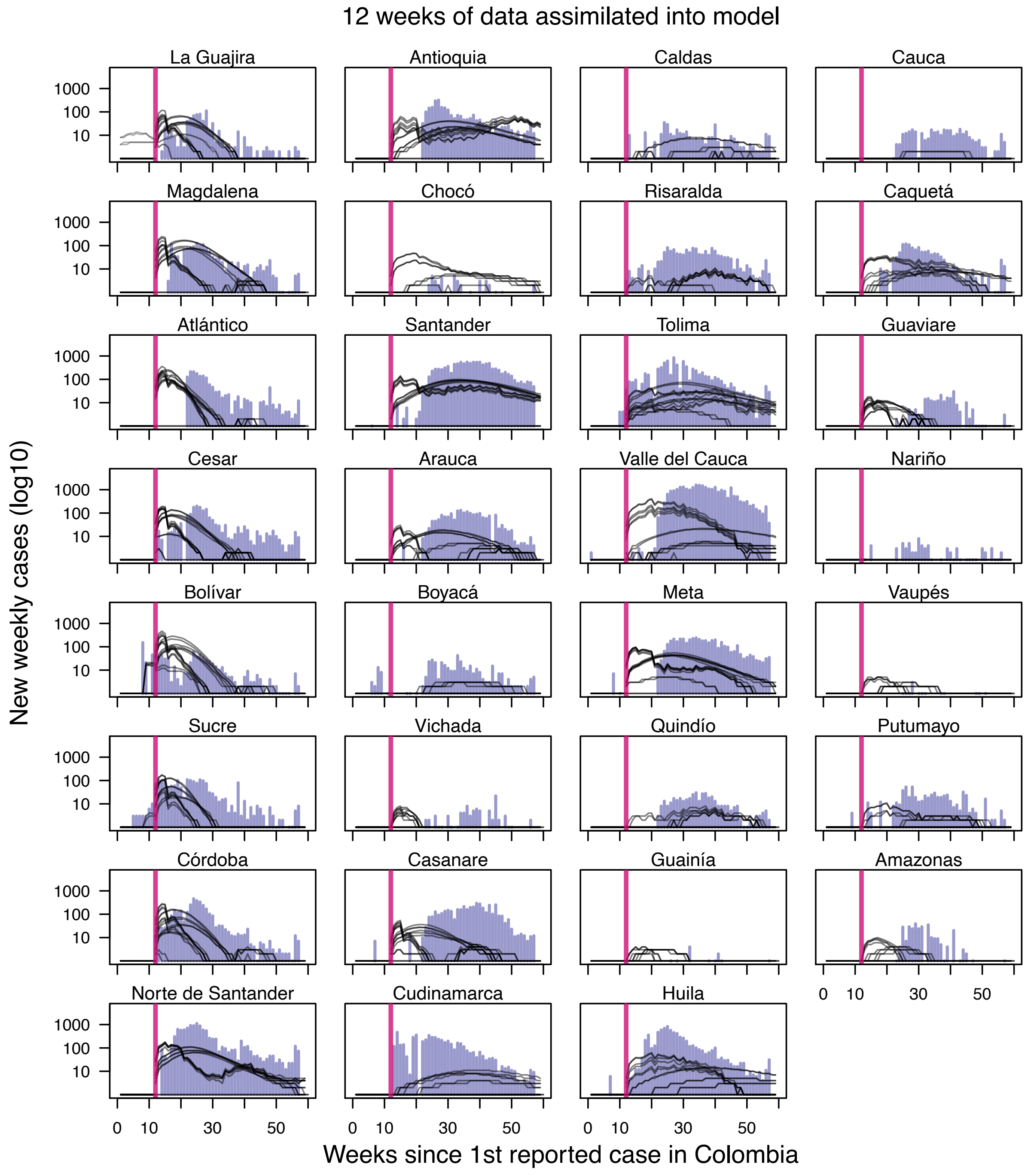

Supplementary Figure 13: **Observed incidence with median forecast for each of the 16 models with 12 weeks of data assimilated into forecasting models.** Navy bars indicate Zika incidence data at weekly time interval [14]. Vertical line indicates the point at which the forecast was made, with data to the left of the line assimilated into the model. Forecasts to the right of the vertical line change as more data is assimilated into the model, while model fits to the left of the vertical line do not change.

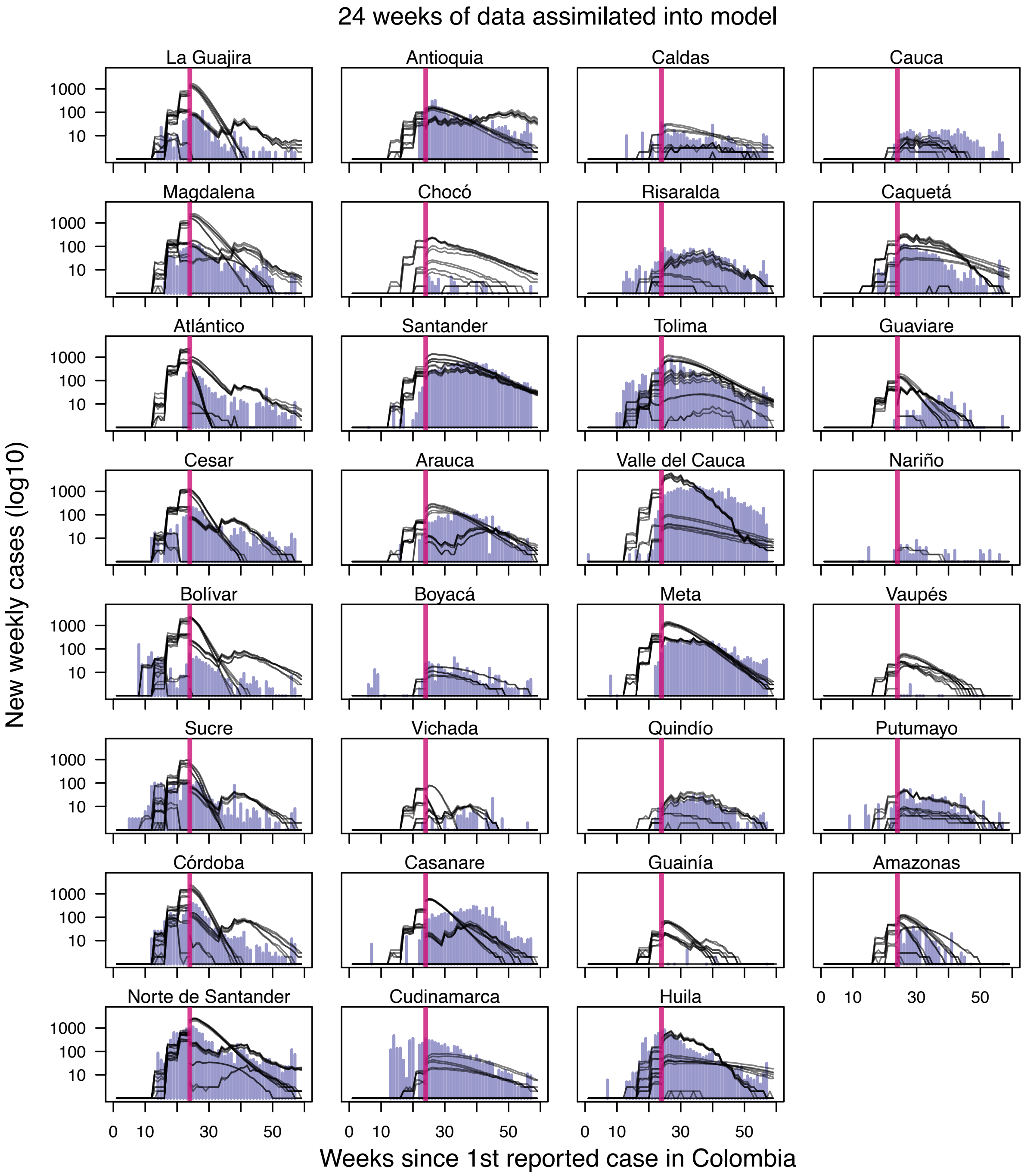

Supplementary Figure 14: **Observed incidence with median forecast for each of the 16 models with 24 weeks of data assimilated into forecasting models.** Navy bars indicate Zika incidence data at weekly time interval [14]. Vertical line indicates the point at which the forecast was made, with data to the left of the line assimilated into the model. Forecasts to the right of the vertical line change as more data is assimilated into the model, while model fits to the left of the vertical line do not change.

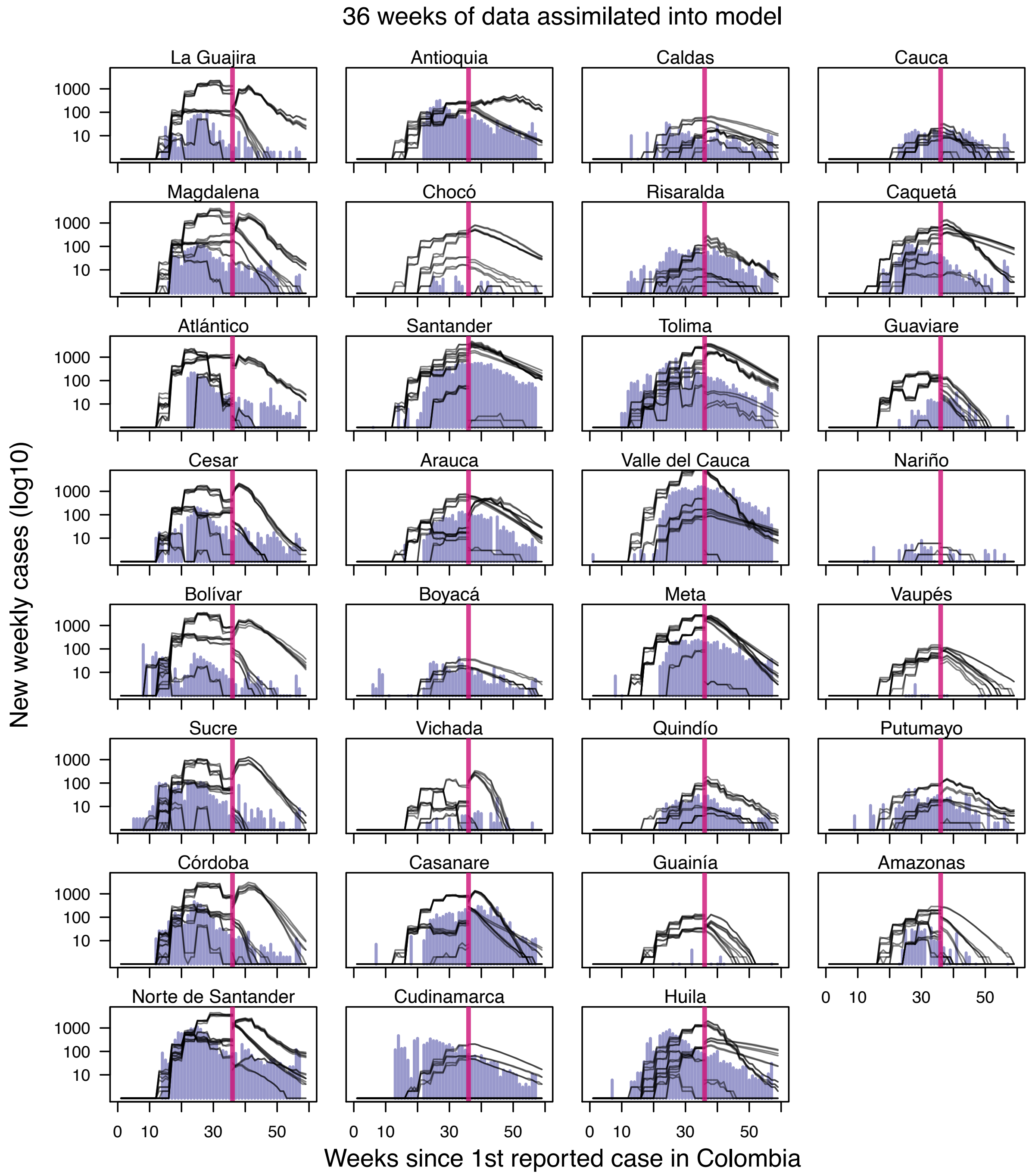

Supplementary Figure 15: **Observed incidence with median forecast for each of the 16 models with 36 weeks of data assimilated into forecasting models.** Navy bars indicate Zika incidence data at weekly time interval [14]. Vertical line indicates the point at which the forecast was made, with data to the left of the line assimilated into the model. Forecasts to the right of the vertical line change as more data is assimilated into the model, while model fits to the left of the vertical line do not change.

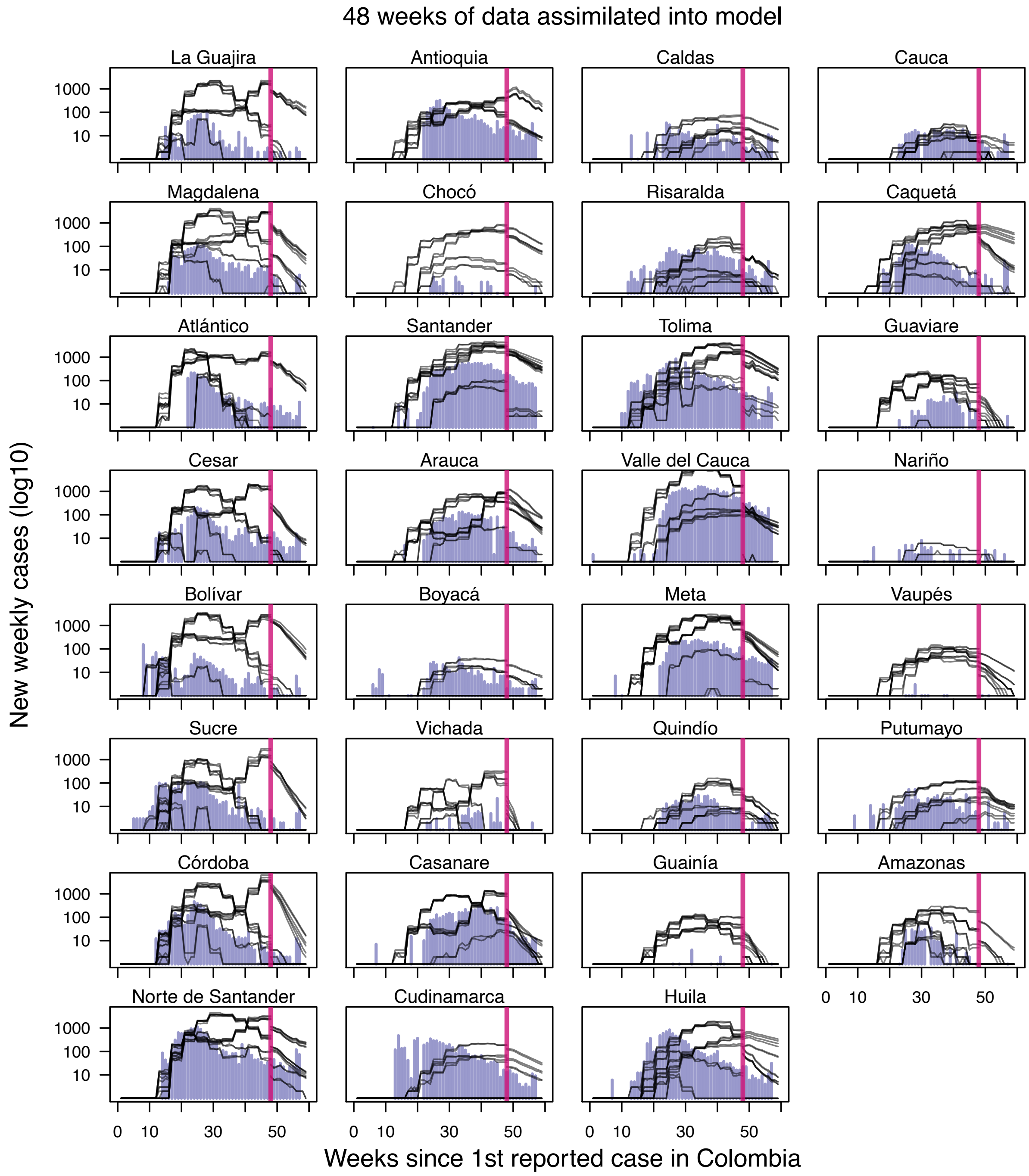

Supplementary Figure 16: **Observed incidence with median forecast for each of the 16 models with 48 weeks of data assimilated into forecasting models.** Navy bars indicate Zika incidence data at weekly time interval [14]. Vertical line indicates the point at which the forecast was made, with data to the left of the line assimilated into the model. Forecasts to the right of the vertical line change as more data is assimilated into the model, while model fits to the left of the vertical line do not change.

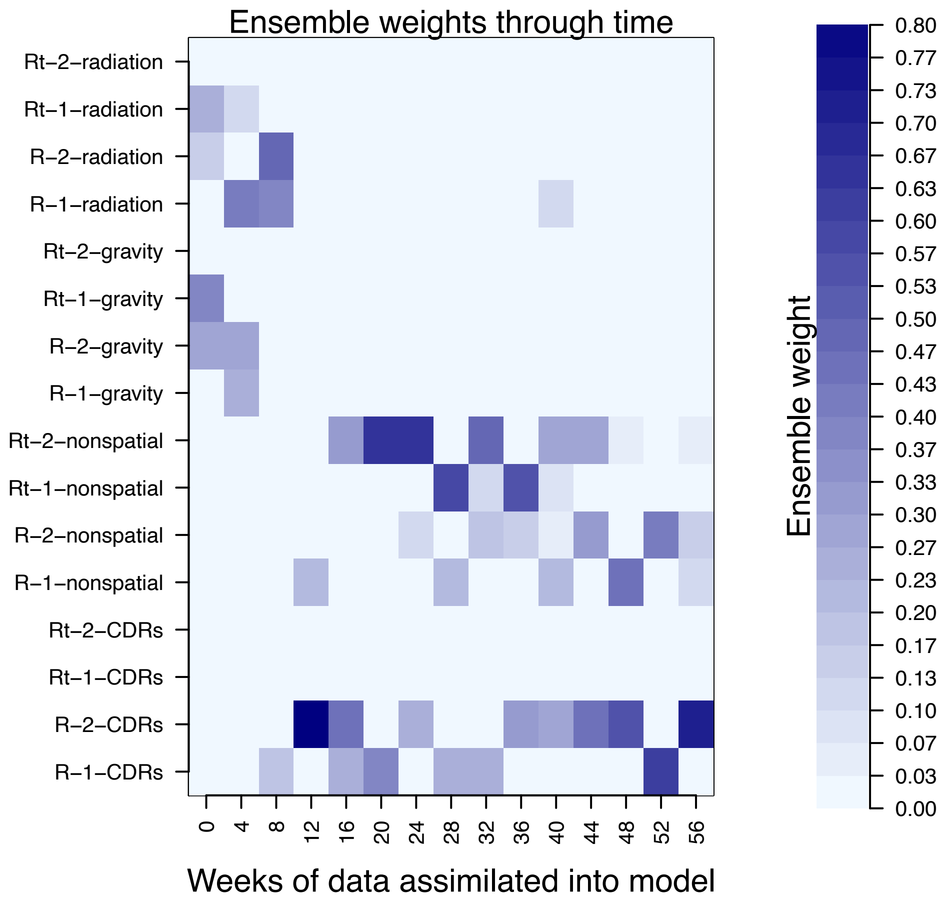

Supplementary Figure 17: **Ensemble weights of each model at each assimilation period.** Ensemble weights were calculated using the expectation-maximization algorithm on short-term forecast performances, where forecast performance was assessed against 4-wk ahead incidence in a given department and nationally.

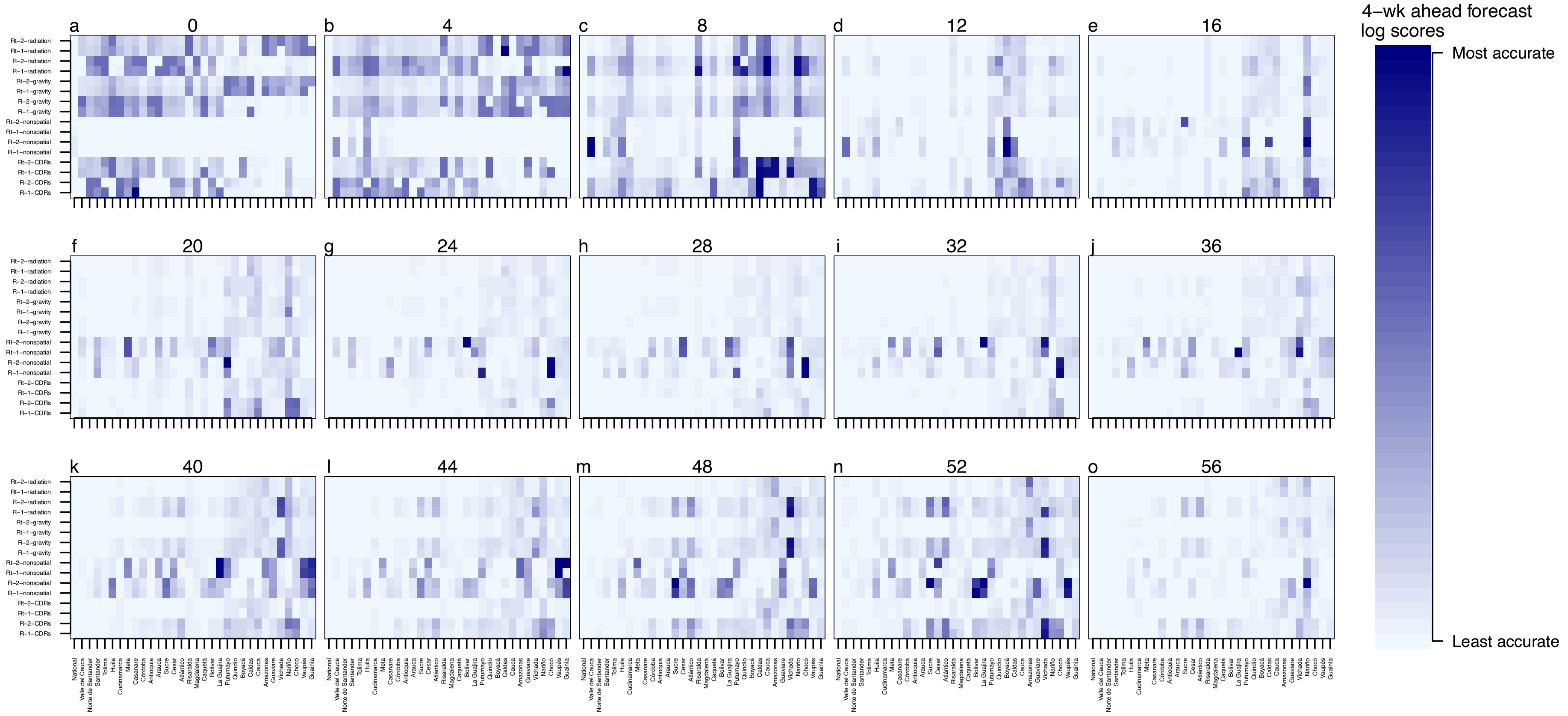

Supplementary Figure 18: **Log scores of each model and department at each assimilation period.** Log scores were assessed using cumulative 4-wk ahead forecasts for each department and aggregated nationally. These log scores were then used in the expectation-maximization algorithm.

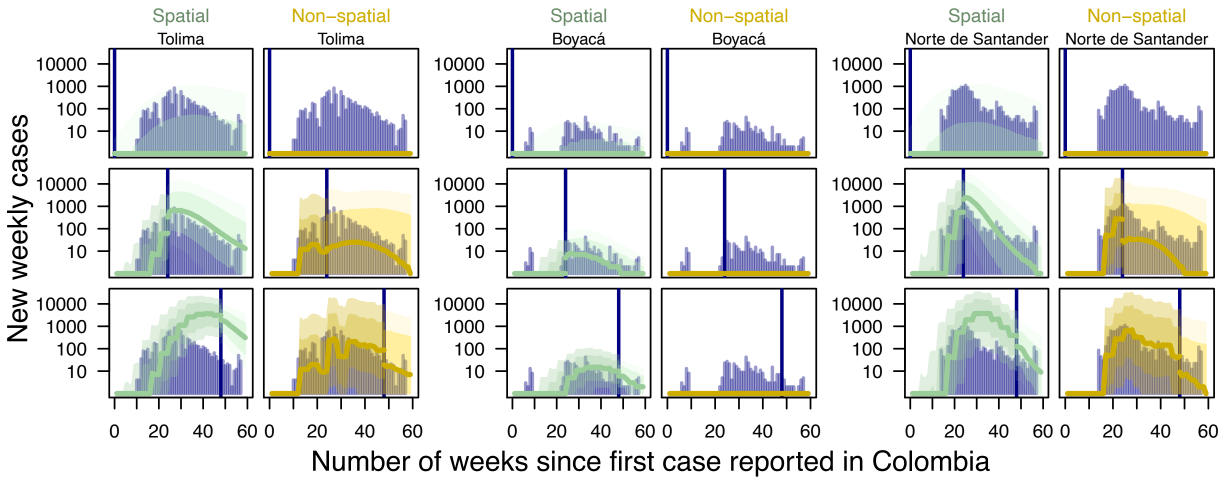

Supplementary Figure 19: **Forecasts for a spatially coupled (green) and uncoupled (yellow) models with one introduction and static R for three departments.** Navy bars denote incidence data. Wide, light bands denote the 75% CrI, narrow, dark band denotes the 50% CrI, and thick line denotes the median forecast. Each row is from the same time point. Time points were chosen to be equally spaced out through the epidemic, with the first set of forecasts from the week of the first case, the second set of forecasts generated at 24 weeks after the first case was reported in Colombia, and the third set of forecasts generated at 48 weeks after the first case was reported in Colombia.

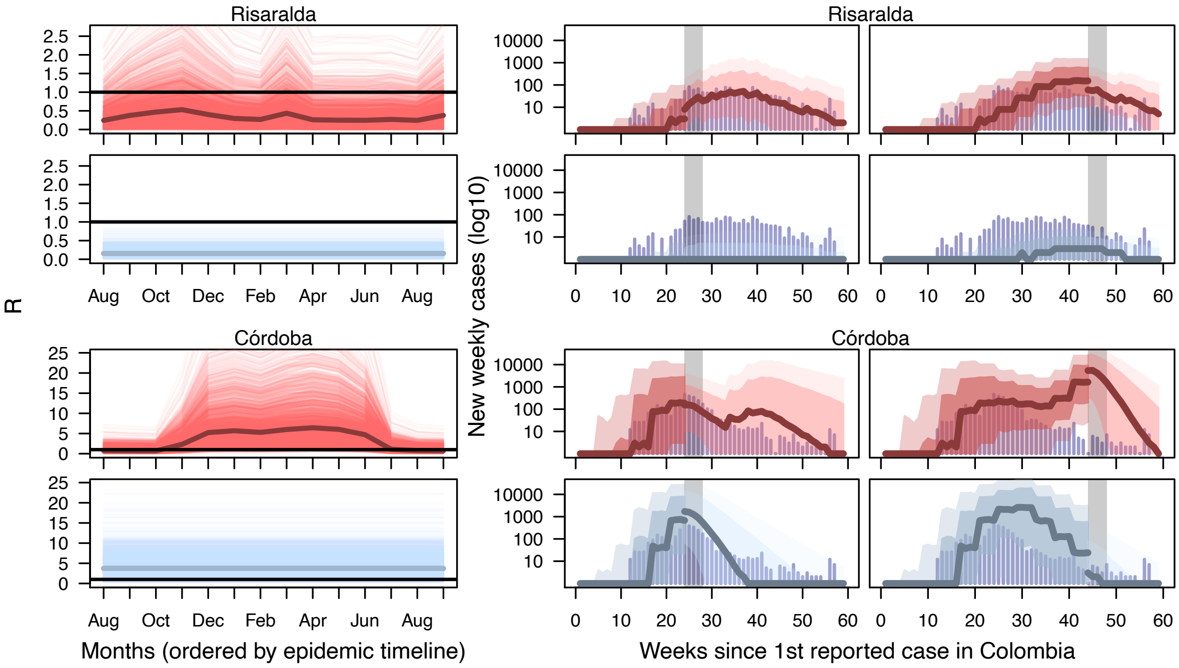

Supplementary Figure 20: **Fitted R and forecasts in two departments for models with two ZIKV introductions, cell phone mobility informed human movement, and a dynamic or static R.** Models with dynamic R are depicted in red and models with a static R are depicted in blue. In plots of R, each line is a draw from the posterior, with a bold median line; horizontal black line depicts $R = 1$. The first set of forecasts in the middle column are from the peak week in both Risaralda and Córdoba, 24-28 weeks after the first case was reported in Colombia, and the second set are from 44-48 weeks after the first case was reported in Colombia. Vertical grey bars depict the forecasts and data considered when assessing short-term forecast performance. Narrow navy bars denote incidence data. Wide, light bands denote the 75% CrI, narrow, dark band denotes the 50% CrI, and thick line denotes the median forecast. Each row is from the same time point.

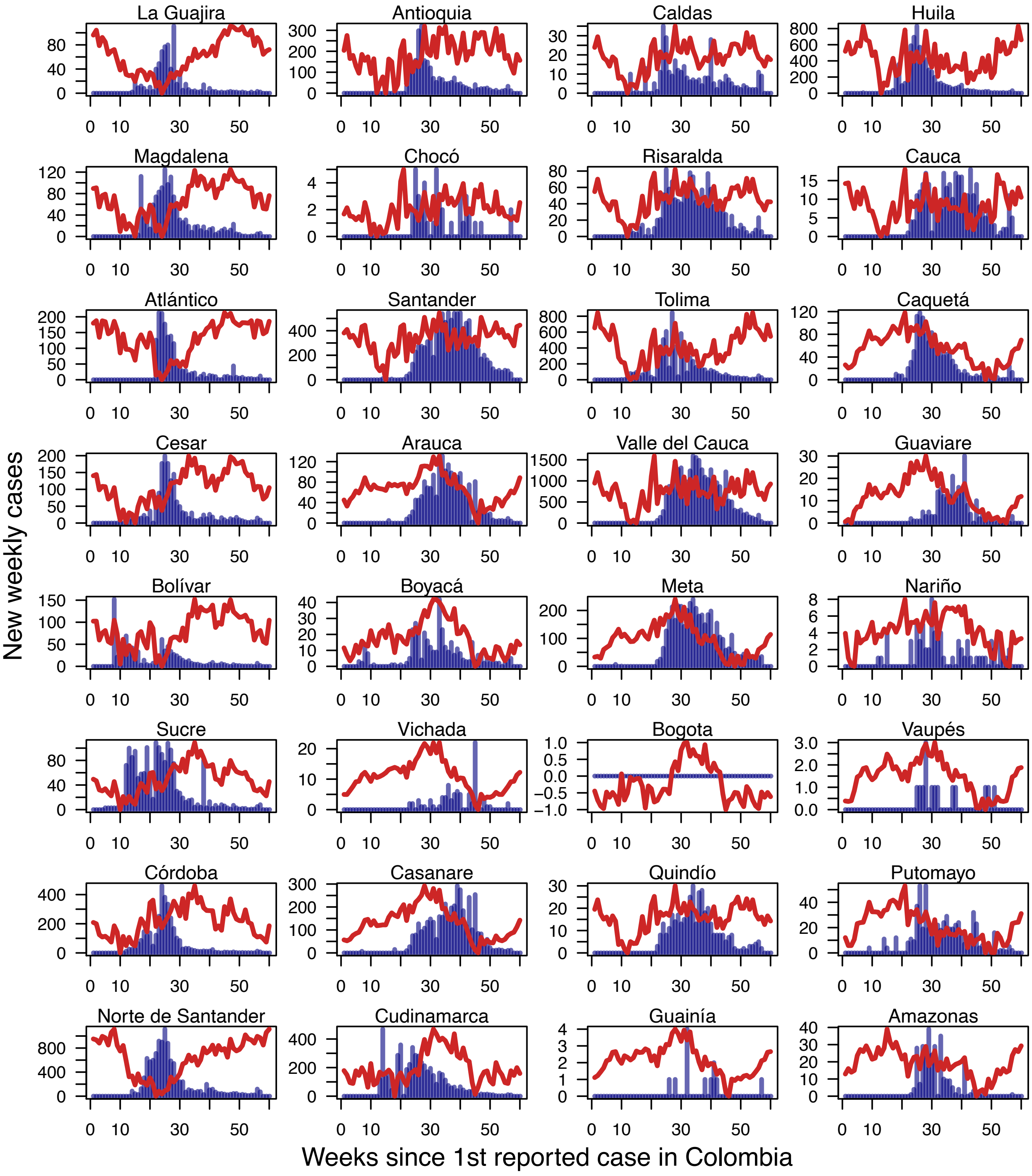

Supplementary Figure 21: **Incidence by department with temperature trends.** Blue bars denote weekly Zika incidence and red line denotes temperature trends.

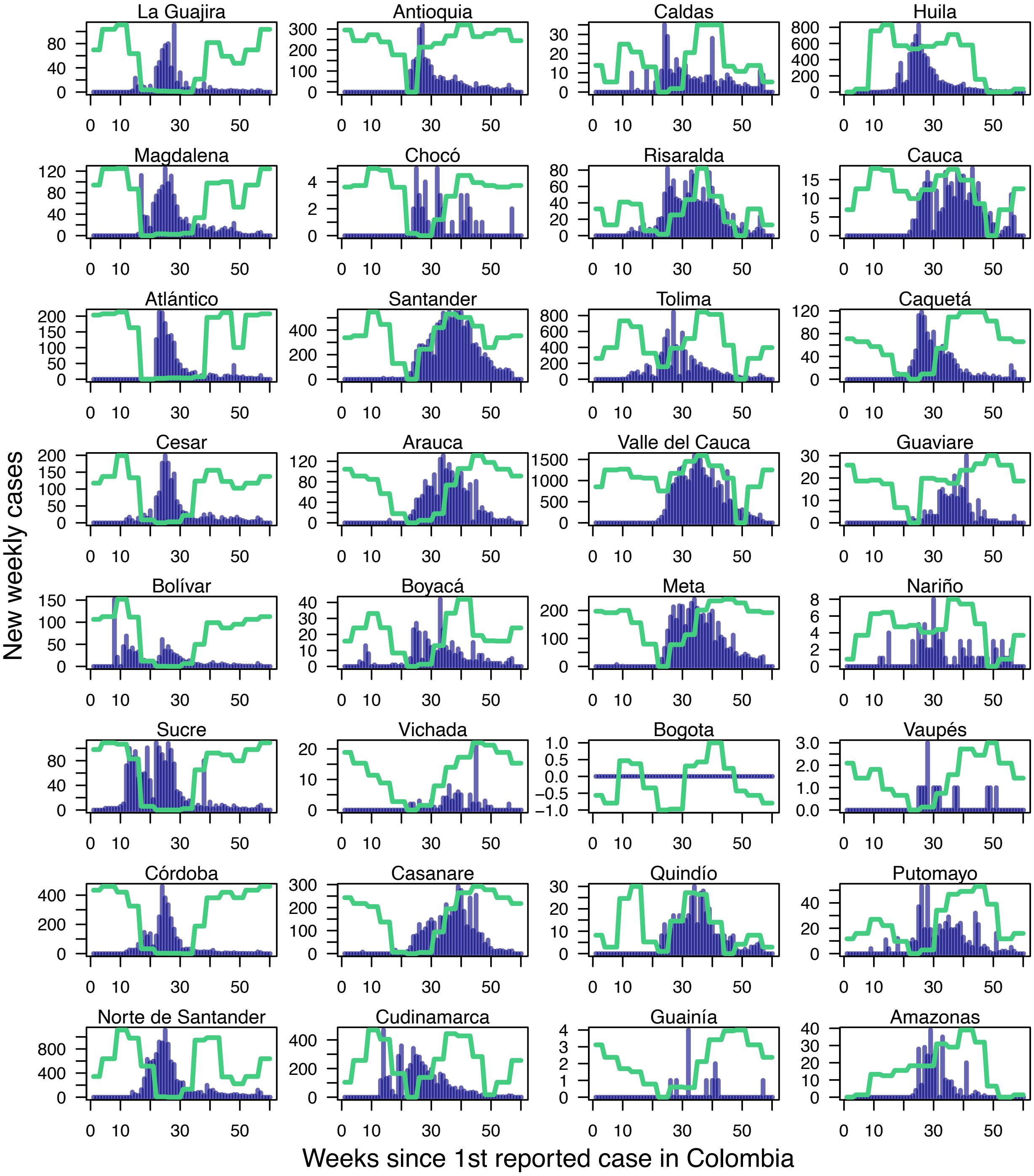

Supplementary Figure 22: **Incidence by department with mosquito occurrence probability trends.** Blue bars denote weekly Zika incidence and green line denotes mosquito occurrence trends.

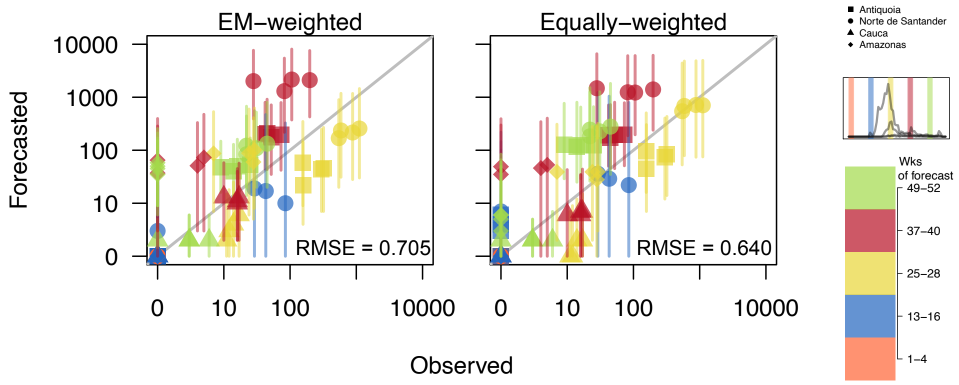

Supplementary Figure 23: **Observed versus forecasted incidence at 1-, 2-, 3-, and 4-week ahead intervals for EM-weighted and equally weighted ensemble models for Antioquia, Norte de Santander, Cauca, and Amazonas.** Plotted departments reflect differences in population, epidemic size, and geographic regions of Colombia and are denoted by point type. Point shape denotes department. Point color indicates time at which the forecast was made (visually denoted in inset plot and color bar). Point is the median value and lines are the 50% credible interval, which were calculated from 2,000 draws from the posterior fit of the model. 1:1 line is in grey. The root mean square errors for the EM-weighted and equally weighted forecasts shown here are 0.705 and 0.640, respectively.

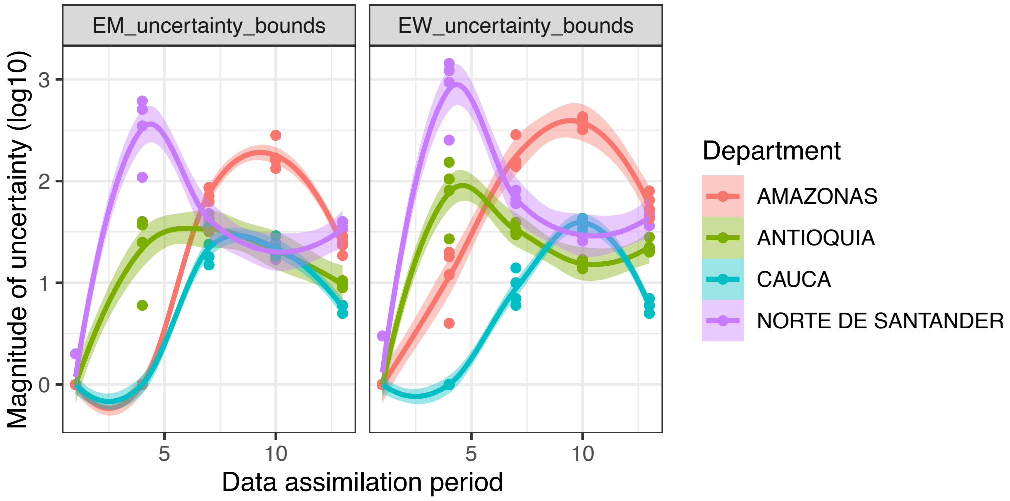

Supplementary Figure 24: **Magnitude of the 50% uncertainty bounds (as shown in Fig. 23) for 1-, 2-, 3-, and 4- week ahead forecasts in five different data assimilation periods for the EM-weighted and equally weighted ensemble models for Amazonas, Antioquia, Cauca, and Norte de Santander.** The four points per data assimilation period represent the 1-, 2-, 3-, and 4-week ahead forecasts for each of the four departments denoted by color. Smoothed loess lines, with error bars representing the standard error, are shown to demonstrate how the magnitude of uncertainty changes through time for each department.

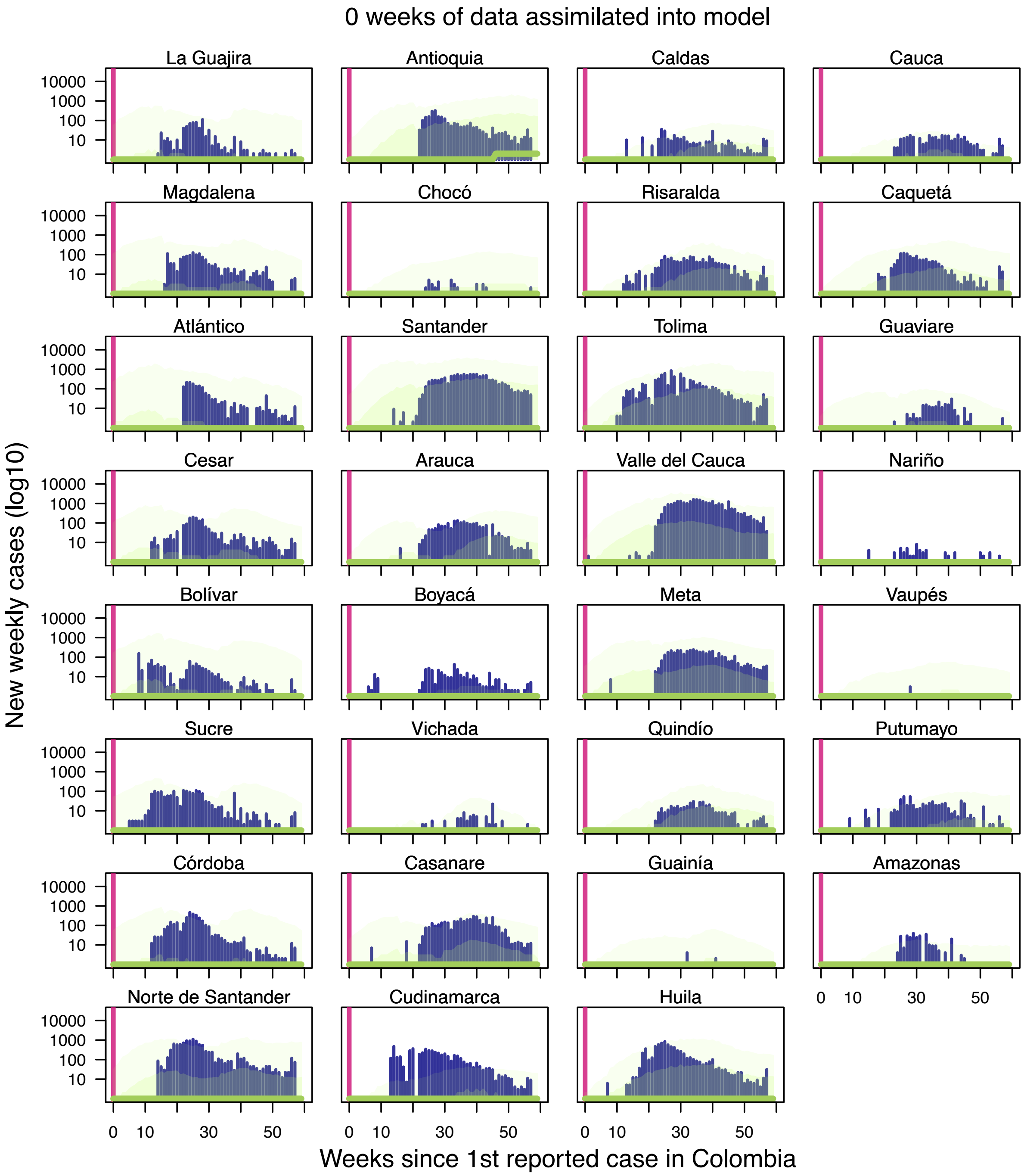

Supplementary Figure 25: **Observed incidence with initial expectation maximization algorithm-weighted ensemble forecast with no weeks of data yet assimilated into forecasting models.** Navy bars indicate Zika incidence data at weekly time interval [14]. Light green band denotes 75% credible interval, darker green band denotes 50% credible interval, and the dark green line denotes median ensemble forecast. Vertical line indicates the point at which the forecast was made, with data to the left of the line assimilated into the model. Forecasts to the right of the vertical line change as more data is assimilated into the model, while model fits to the left of the vertical line do not change.

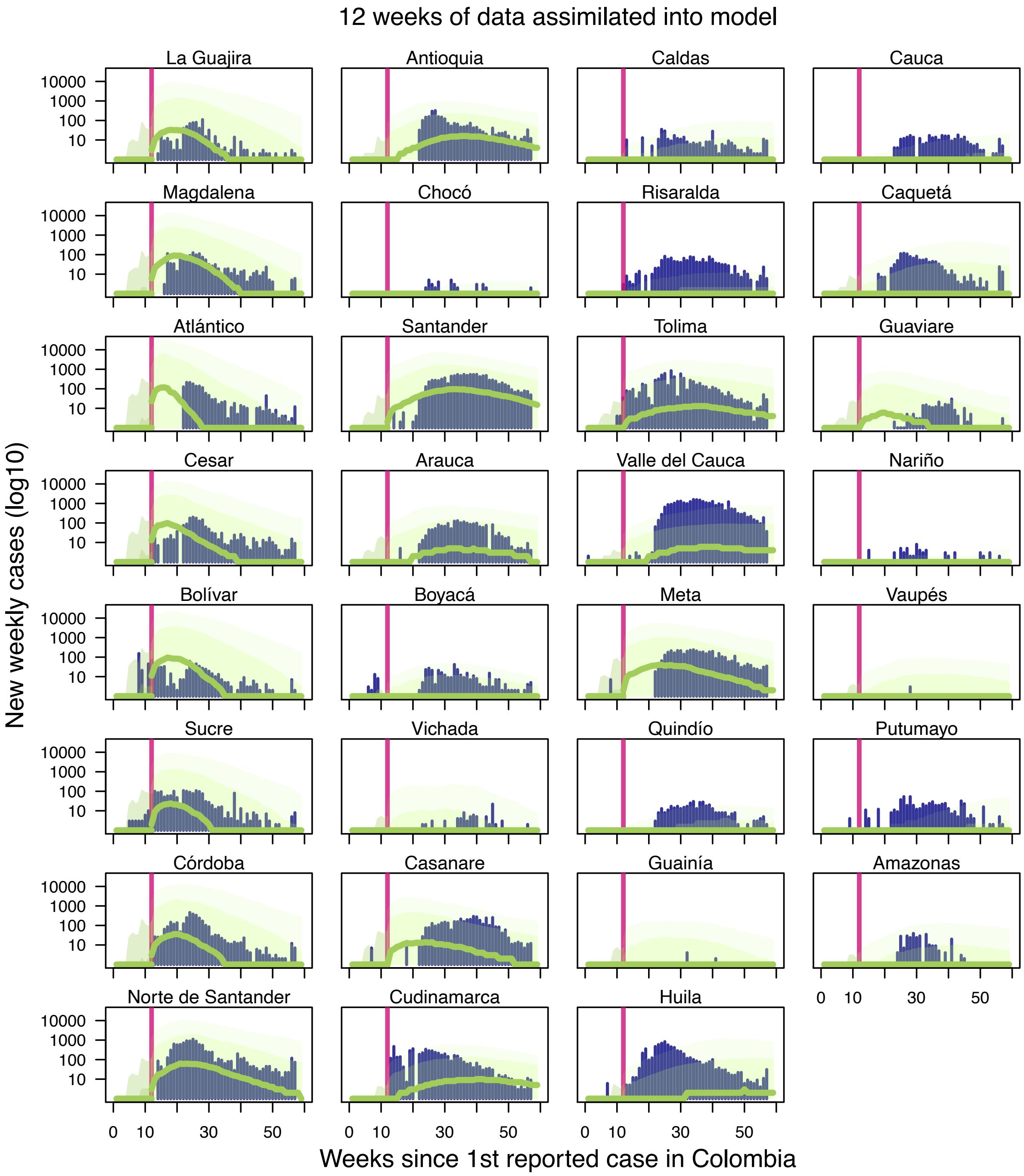

Supplementary Figure 26: **Observed incidence with expectation maximization algorithm-weighted ensemble forecast with 12 weeks of data assimilated into forecasting models.** Navy bars indicate Zika incidence data at weekly time interval [14]. Light green band denotes 75% credible interval, darker green band denotes 50% credible interval, and the dark green line denotes median ensemble forecast. Vertical line indicates the point at which the forecast was made, with data to the left of the line assimilated into the model. Forecasts to the right of the vertical line change as more data is assimilated into the model, while model fits to the left of the vertical line do not change.

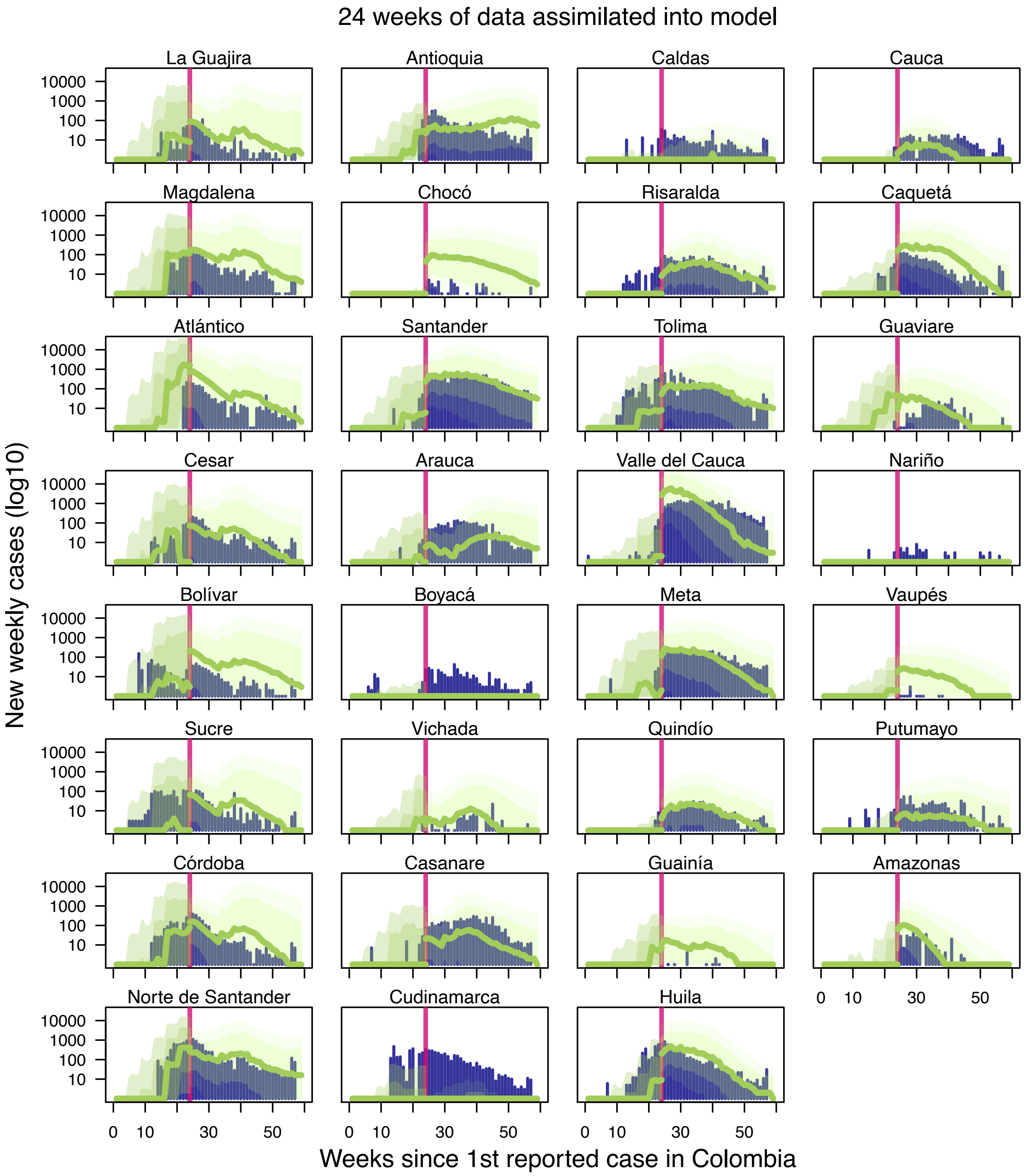

Supplementary Figure 27: **Observed incidence with expectation maximization algorithm-weighted ensemble forecast with 24 weeks of data assimilated into forecasting models.** Navy bars indicate Zika incidence data at weekly time interval [14]. Light green band denotes 75% credible interval, darker green band denotes 50% credible interval, and the dark green line denotes median ensemble forecast. Vertical line indicates the point at which the forecast was made, with data to the left of the line assimilated into the model. Forecasts to the right of the vertical line change as more data is assimilated into the model, while model fits to the left of the vertical line do not change.

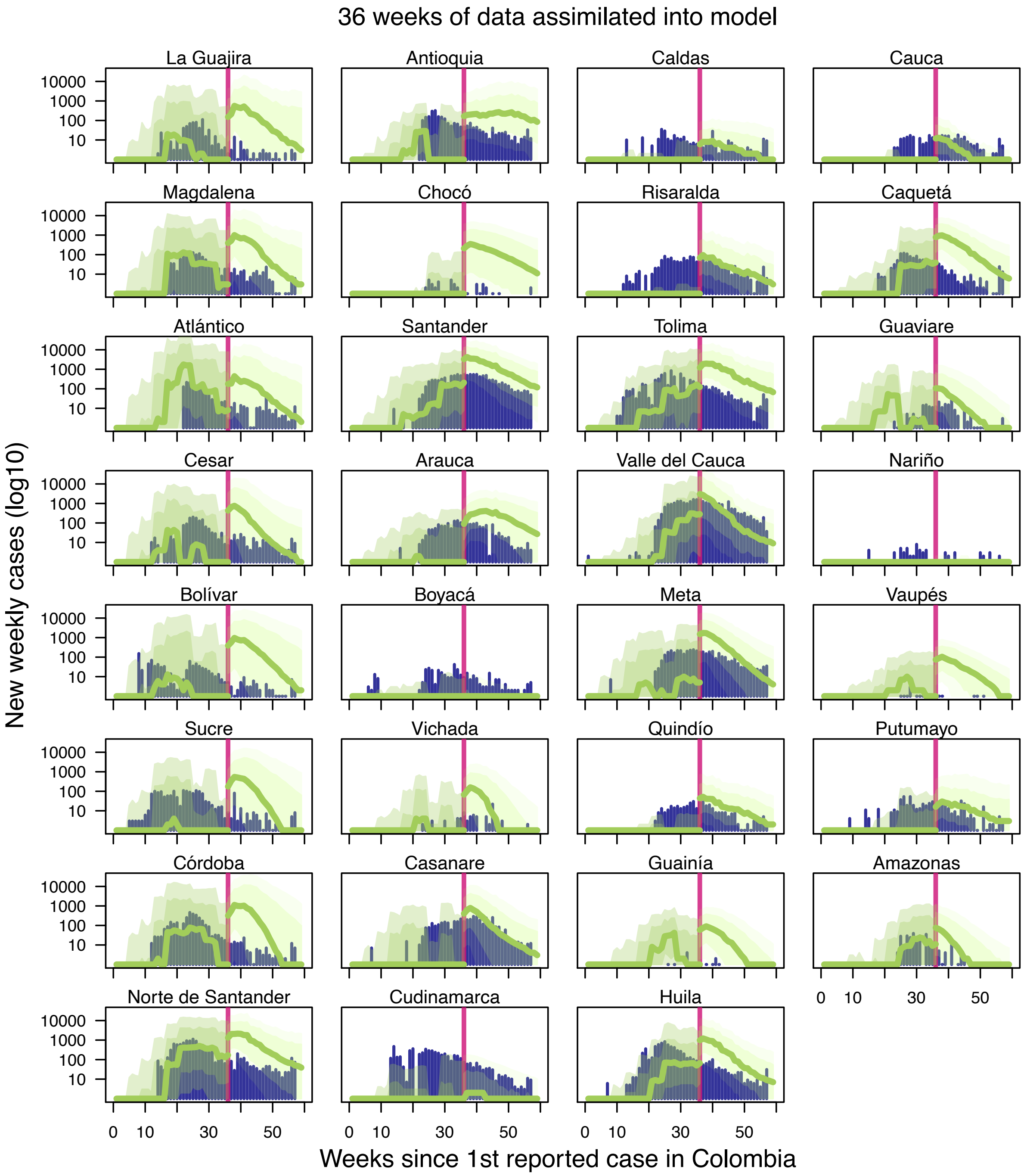

Supplementary Figure 28: **Observed incidence with expectation maximization algorithm-weighted ensemble forecast with 36 weeks of data assimilated into forecasting models.** Navy bars indicate Zika incidence data at weekly time interval [14]. Light green band denotes 75% credible interval, darker green band denotes 50% credible interval, and the dark green line denotes median ensemble forecast. Vertical line indicates the point at which the forecast was made, with data to the left of the line assimilated into the model. Forecasts to the right of the vertical line change as more data is assimilated into the model, while model fits to the left of the vertical line do not change.

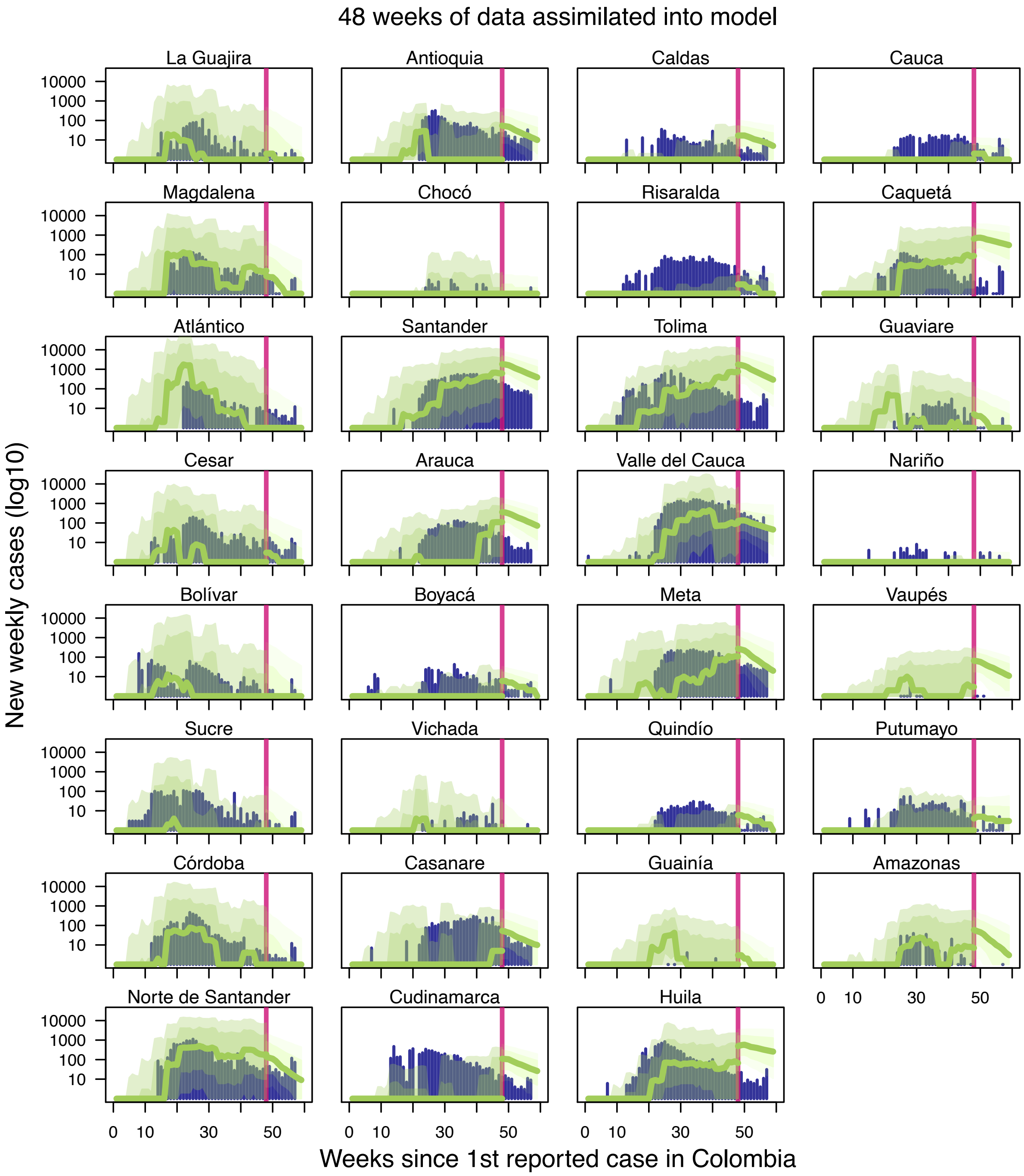

Supplementary Figure 29: **Observed incidence with expectation maximization algorithm-weighted ensemble forecast with 48 weeks of data assimilated into forecasting models.** Navy bars indicate Zika incidence data at weekly time interval [14]. Light green band denotes 75% credible interval, darker green band denotes 50% credible interval, and the dark green line denotes median ensemble forecast. Vertical line indicates the point at which the forecast was made, with data to the left of the line assimilated into the model. Forecasts to the right of the vertical line change as more data is assimilated into the model, while model fits to the left of the vertical line do not change.

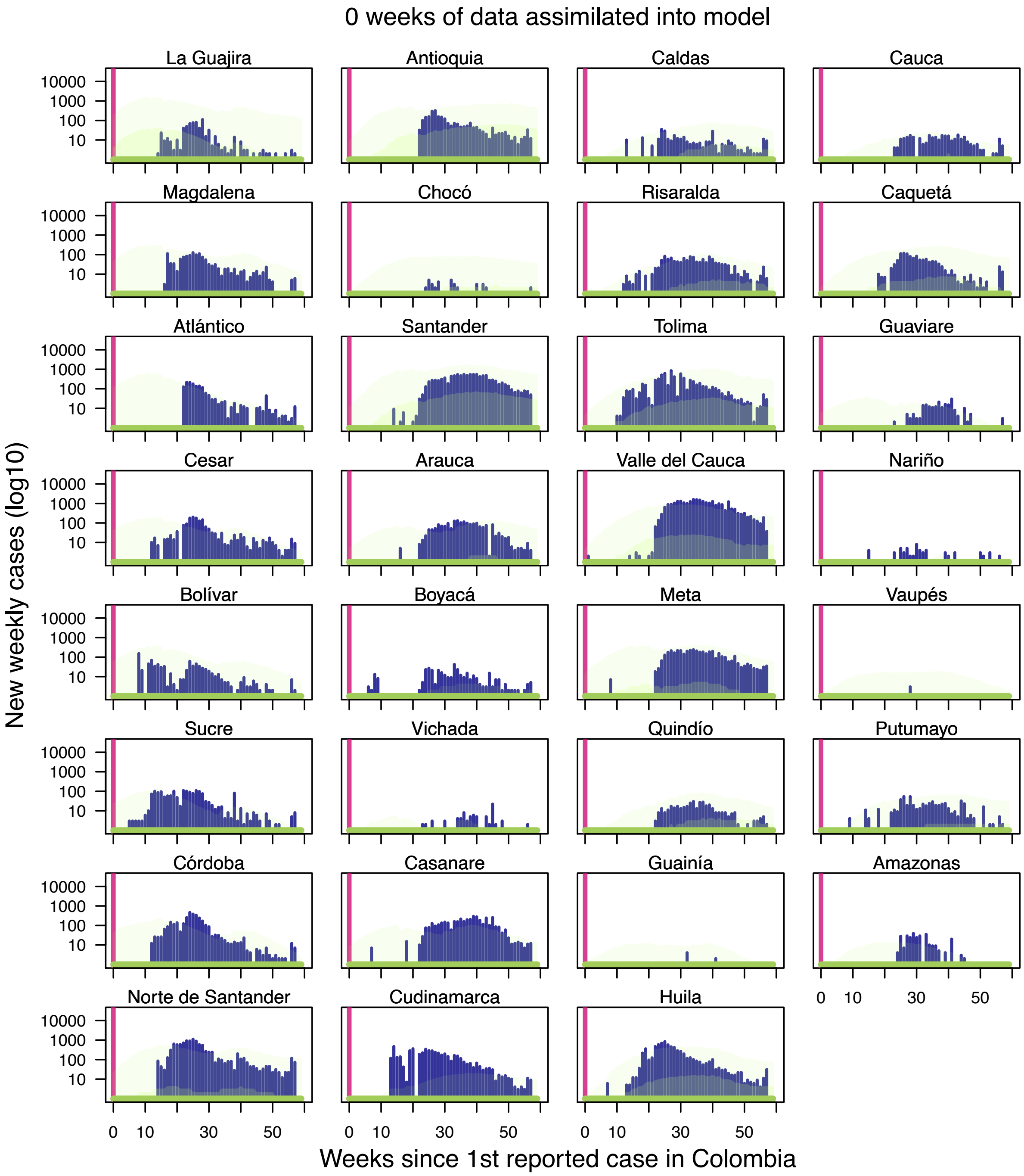

Supplementary Figure 30: **Observed incidence with initial equally weighted ensemble forecast with no weeks of data yet assimilated into forecasting models.** Navy bars indicate Zika incidence data at weekly time interval [14]. Light green band denotes 75% credible interval, darker green band denotes 50% credible interval, and the dark green line denotes median ensemble forecast. Vertical line indicates the point at which the forecast was made, with data to the left of the line assimilated into the model. Forecasts to the right of the vertical line change as more data is assimilated into the model, while model fits to the left of the vertical line do not change.

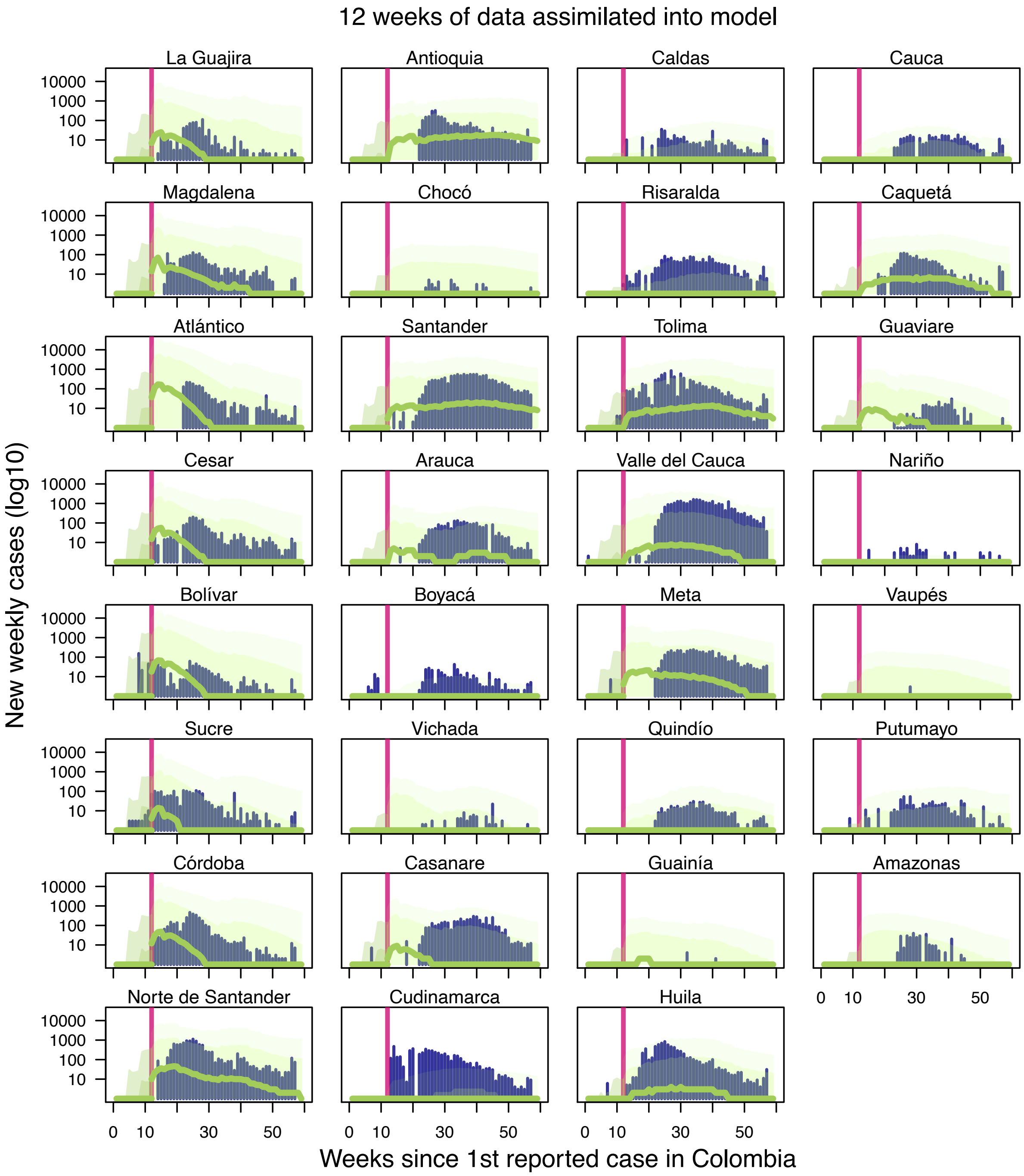

Supplementary Figure 31: **Observed incidence with equally weighted ensemble forecast with 12 weeks of data assimilated into forecasting models.** Navy bars indicate Zika incidence data at weekly time interval [14]. Light green band denotes 75% credible interval, darker green band denotes 50% credible interval, and the dark green line denotes median ensemble forecast. Vertical line indicates the point at which the forecast was made, with data to the left of the line assimilated into the model. Forecasts to the right of the vertical line change as more data is assimilated into the model, while model fits to the left of the vertical line do not change.

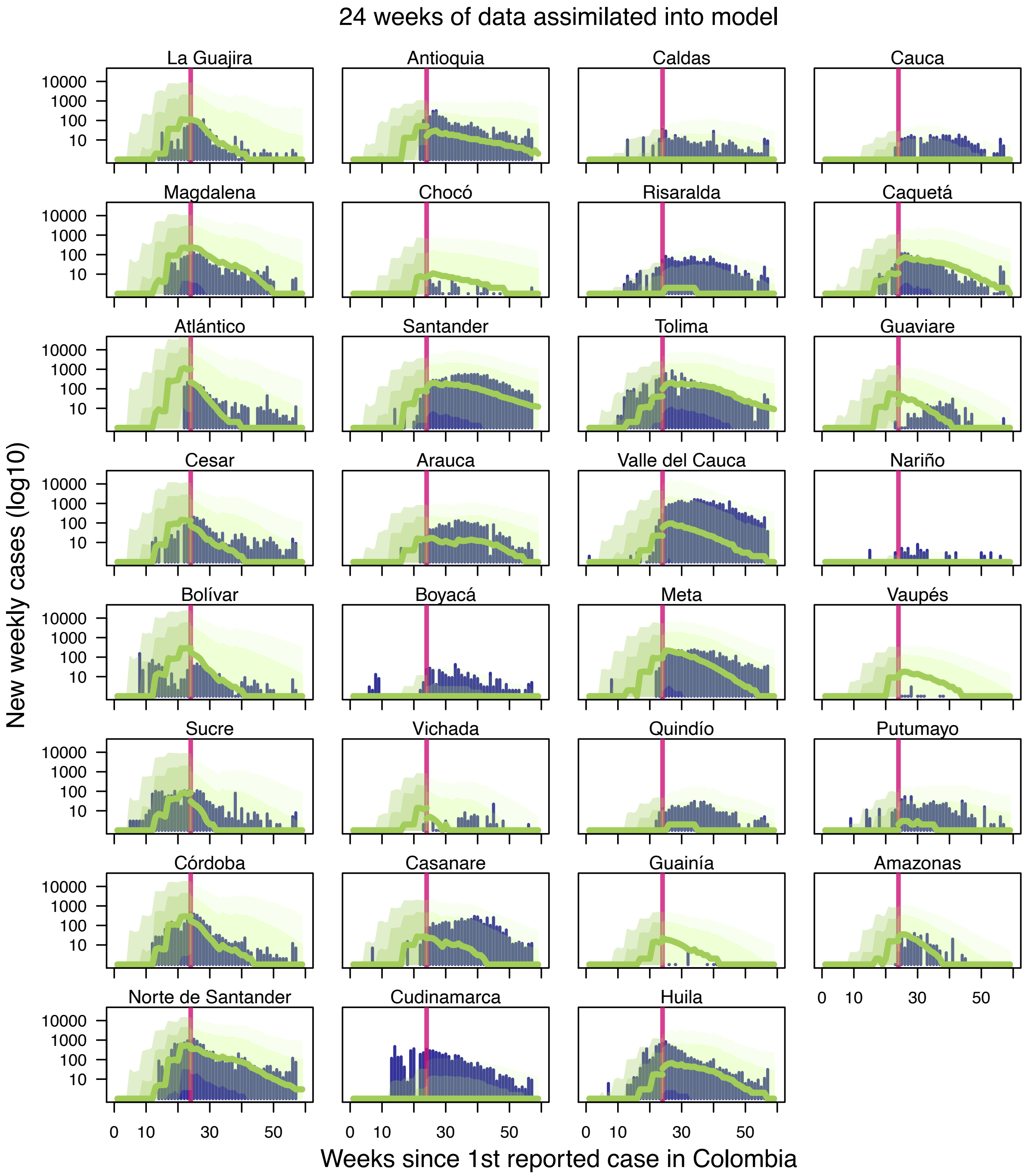

Supplementary Figure 32: **Observed incidence with equally weighted ensemble forecast with 24 weeks of data assimilated into forecasting models.** Navy bars indicate Zika incidence data at weekly time interval [14]. Light green band denotes 75% credible interval, darker green band denotes 50% credible interval, and the dark green line denotes median ensemble forecast. Vertical line indicates the point at which the forecast was made, with data to the left of the line assimilated into the model. Forecasts to the right of the vertical line change as more data is assimilated into the model, while model fits to the left of the vertical line do not change.

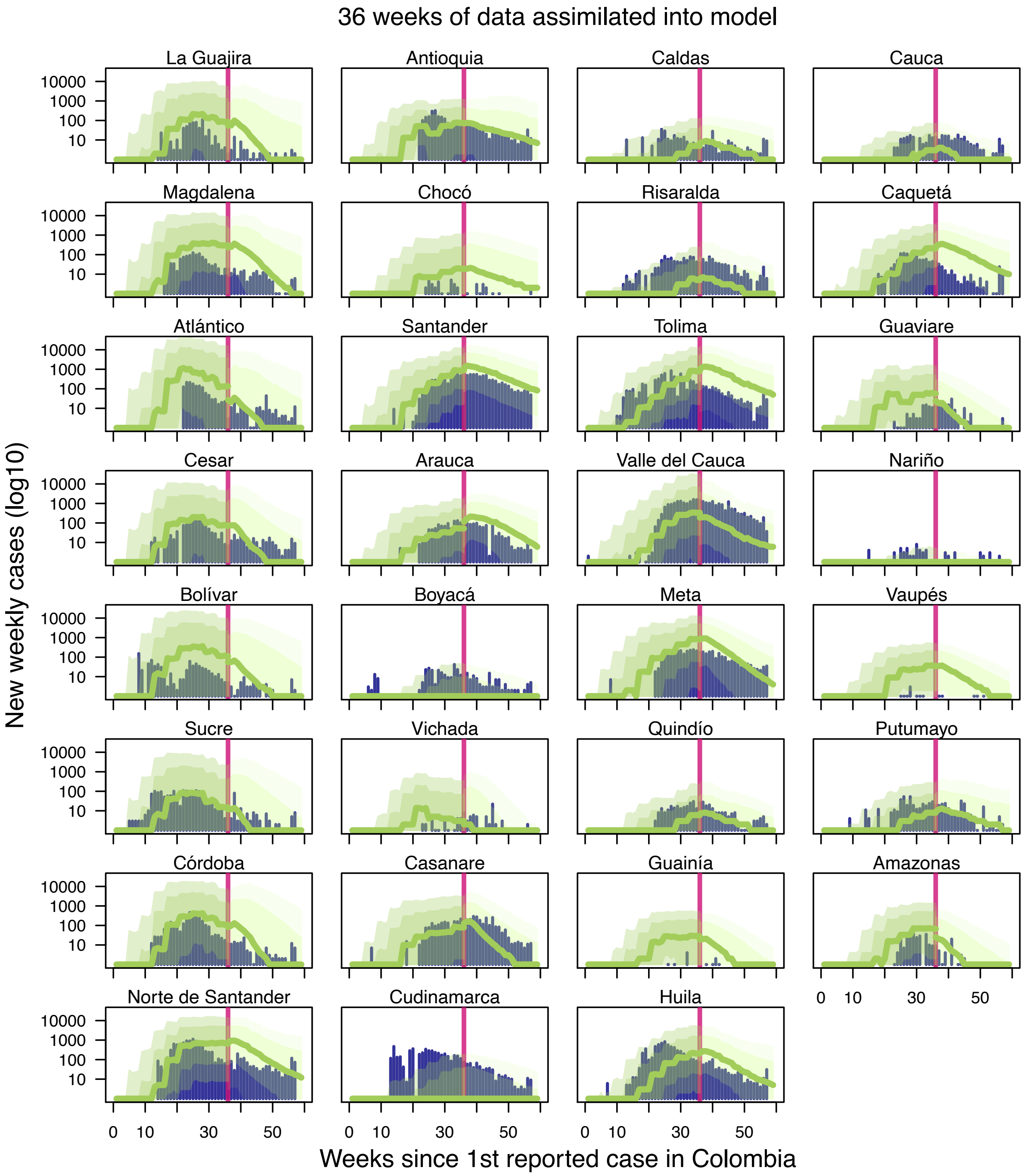

Supplementary Figure 33: **Observed incidence with equally weighted ensemble forecast with 36 weeks of data assimilated into forecasting models.** Navy bars indicate Zika incidence data at weekly time interval [14]. Light green band denotes 75% credible interval, darker green band denotes 50% credible interval, and the dark green line denotes median ensemble forecast. Vertical line indicates the point at which the forecast was made, with data to the left of the line assimilated into the model. Forecasts to the right of the vertical line change as more data is assimilated into the model, while model fits to the left of the vertical line do not change.

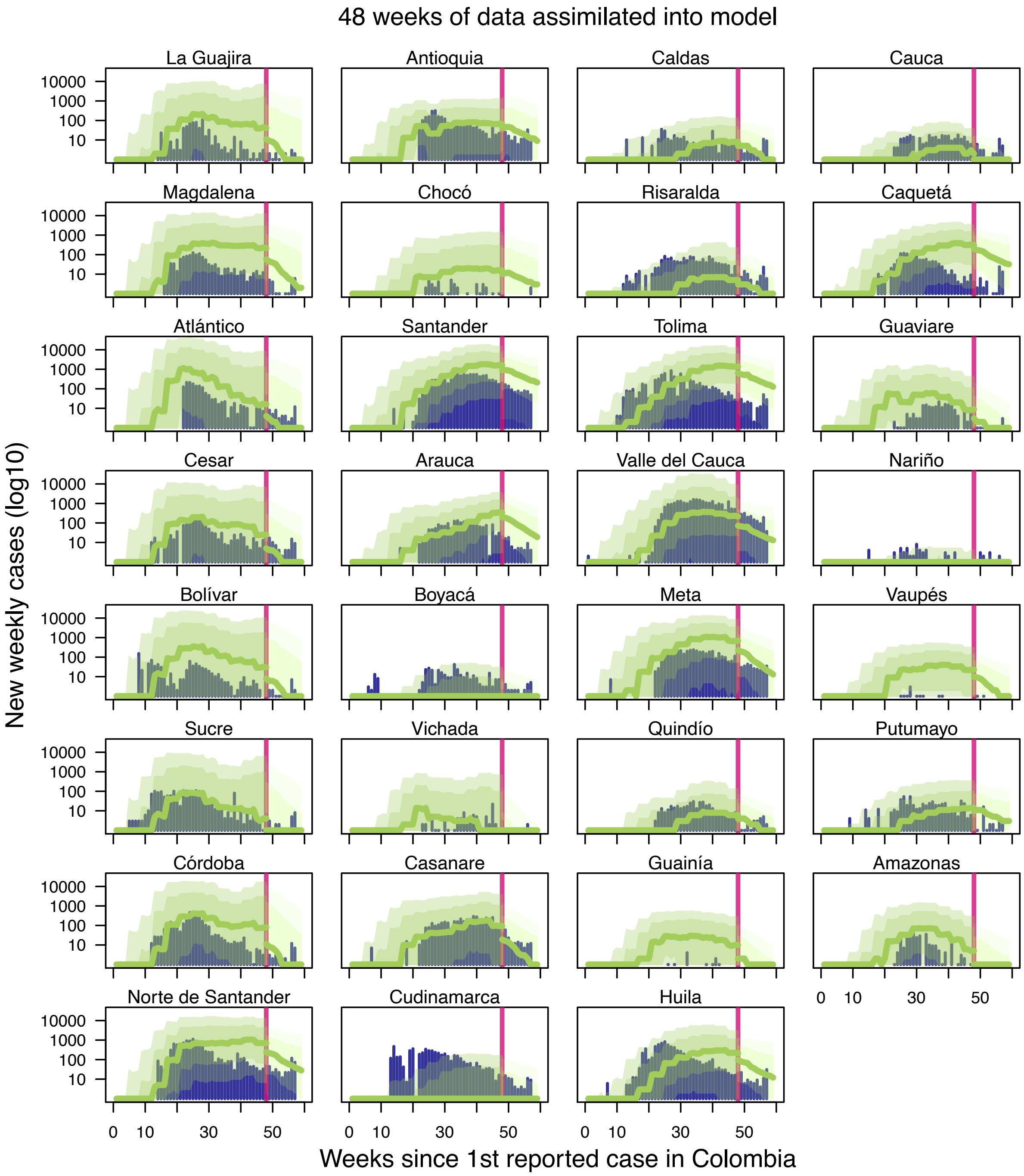

Supplementary Figure 34: **Observed incidence with equally weighted ensemble forecast with 48 weeks of data assimilated into forecasting models.** Navy bars indicate Zika incidence data at weekly time interval [14]. Light green band denotes 75% credible interval, darker green band denotes 50% credible interval, and the dark green line denotes median ensemble forecast. Vertical line indicates the point at which the forecast was made, with data to the left of the line assimilated into the model. Forecasts to the right of the vertical line change as more data is assimilated into the model, while model fits to the left of the vertical line do not change.

References

[1] Sebastian Funk et al. "Comparative Analysis of Dengue and Zika Outbreaks Reveals Differences by Setting and Virus". In: *PLOS Neglected Tropical Diseases* 10.12 (Dec. 2016), pp. 1–16. DOI: 10.1371/journal.pntd.0005173. URL: https://doi.org/10.1371/journal.pntd.0005173.

[2] Samir Bhatt et al. "The global distribution and burden of dengue". In: *Nature* 496.7446 (Apr. 2013), pp. 504–507. ISSN: 1476-4687. DOI: 10.1038/nature12060. URL: https://doi.org/10.1038/nature12060.

[3] Monaise M.O. Silva et al. "Accuracy of dengue reporting by national surveillance system, Brazil". In: *Emerging Infectious Diseases* 22.2 (Feb. 2016). DOI: https://dx.doi.org/10.3201/eid2202.150495. URL: https://wwwnc.cdc.gov/eid/article/22/2/15-0495_article.

[4] Deborah P. Shutt et al. "Estimating the reproductive number, total outbreak size, and reporting rates for Zika epidemics in South and Central America". In: *Epidemics* 21 (Dec. 2017), pp. 63–79. ISSN: 1755-4365. URL: http://www.sciencedirect.com/science/article/pii/S1755436517300257.

[5] Rachel J Oidtman, Guido España, and T Alex Perkins. "Co-circulation and misdiagnosis led to underestimation of the 2015-2017 Zika epidemic in the Americas". In: *medRxiv* (2020). DOI: https://doi.org/10.1101/19010256. URL: https://www.medrxiv.org/content/10.1101/19010256v1.

[6] Sean M. Moore et al. "Leveraging multiple data types to estimate the size of the Zika epidemic in the Americas". In: *PLOS Neglected Tropical Diseases* 14.9 (Sept. 2020), pp. 1–25. DOI: 10.1371/journal.pntd.0008640. URL: https://doi.org/10.1371/journal.pntd.0008640.

[7] M. U. G. Kraemer et al. "Utilizing general human movement models to predict the spread of emerging infectious diseases in resource poor settings". In: *Scientific Reports* 9.1 (2019), p. 5151. ISSN: 2045-2322. DOI: 10.1038/s41598-019-41192-3.

[8] T. Alex Perkins et al. "Model-based projections of Zika virus infections in childbearing women in the Americas". In: *Nature Microbiology* 1.9 (2016), p. 16126. DOI: 10.1038/nmicrobiol.2016.126. URL: https://doi.org/10.1038/nmicrobiol.2016.126.

[9] Erin A. Mordecai et al. "Detecting the impact of temperature on transmission of Zika, dengue, and chikungunya using mechanistic models". In: *PLOS Neglected Tropical Diseases* 11.4 (Apr. 2017), pp. 1–18. DOI: 10.1371/journal.pntd.0005568. URL: https://doi.org/10.1371/journal.pntd.0005568.

[10] Moritz UG Kraemer et al. "The global distribution of the arbovirus vectors *Aedes aegypti* and *Ae. albopictus*". In: *eLife* 4 (June 2015), e08347. ISSN: 2050-084X. DOI: 10.7554/eLife.08347. URL: https://doi.org/10.7554/eLife.08347.

[11] Oliver J. Brady et al. "Global temperature constraints on Aedes aegypti and Ae. albopictus persistence and competence for dengue virus transmission". In: *Parasites & Vectors* 7.1 (July 2014), p. 338. ISSN: 1756-3305. DOI: 10.1186/1756-3305-7-338. URL: https://doi.org/10.1186/1756-3305-7-338.

[12] Miranda Chan and Michael A. Johansson. "The Incubation Periods of Dengue Viruses". In: *PLOS ONE* 7.11 (Nov. 2012), pp. 1–7. DOI: 10.1371/journal.pone.0050972. URL: https://doi.org/10.1371/journal.pone.0050972.

[13] Allison Black et al. "Genomic epidemiology supports multiple introductions and cryptic transmission of Zika virus in Colombia". In: *BMC Infectious Diseases* 19.1 (2019), p. 963. DOI: 10.1186/s12879-019-4566-2. URL: https://doi.org/10.1186/s12879-019-4566-2.

[14] Amir S. Siraj et al. "Spatiotemporal incidence of Zika and associated environmental drivers for the 2015-2016 epidemic in Colombia". In: *Scientific Data* 5.1 (2018), p. 180073. DOI: 10.1038/sdata.2018.73. URL: https://doi.org/10.1038/sdata.2018.73.
